# ^63^Cu(I) binding to human kidney ^68^Zn_7_-βα MT1A: determination of Cu(I)-thiolate cluster domain specificity from ESI-MS and room temperature phosphorescence spectroscopy

**DOI:** 10.1093/mtomcs/mfac101

**Published:** 2022-12-30

**Authors:** Adyn Melenbacher, Lina Heinlein, Andrea Hartwig, Martin J Stillman

**Affiliations:** Department of Chemistry, The University of Western Ontario, 1151 Richmond St., London, Ontario, ON N6A 5B7, Canada; Department of Chemistry, The University of Western Ontario, 1151 Richmond St., London, Ontario, ON N6A 5B7, Canada; Department of Food Chemistry and Toxicology, Institute of Applied Biosciences (IAB), Karlsruhe Institute of Technology (KIT), Adenauerring 20a, Karlsruhe, Baden-Württemberg, 76131, Germany; Department of Food Chemistry and Toxicology, Institute of Applied Biosciences (IAB), Karlsruhe Institute of Technology (KIT), Adenauerring 20a, Karlsruhe, Baden-Württemberg, 76131, Germany; Department of Chemistry, The University of Western Ontario, 1151 Richmond St., London, Ontario, ON N6A 5B7, Canada

**Keywords:** copper homeostasis, electrospray ionization mass spectrometry, emission spectroscopy, metallothionein, metalloprotein, zinc homeostasis

## Abstract

Mammalian metallothioneins (MTs) are important proteins in Zn(II) and Cu(I) homeostasis with the Zn(II) and Cu(I) binding to the 20 cysteines in metal-thiolate clusters. Previous electrospray ionization (ESI) mass spectrometric (MS) analyses of Cu(I) binding to Zn_7_-MT were complicated by significant overlap of the natural abundance isotopic patterns for Zn(II) and Cu(I) leading to impossibly ambiguous stoichiometries. In this paper, isotopically pure ^63^Cu(I) and ^68^Zn(II) allowed determination of the specific stoichiometries in the ^68^ Zn,^63^Cu-βα MT1A species formed following the stepwise addition of ^63^Cu(I) to ^68^Zn_7_-βα MT1A. These species were characterized by ESI-MS and room temperature emission spectroscopy. The key species that form and their emission band centres are Zn_5_Cu_5_-βα MT1A (λ = 684 nm), Zn_4_Cu_6_-βα MT1A (λ = 750 nm), Zn_3_Cu_9_-βα MT1A (λ = 750 nm), Zn_2_Cu_10_-βα MT1A (λ = 750 nm), and Zn_1_Cu_14_-βα MT1A (λ = 634 nm). The specific domain stoichiometry of each species was determined by assessing the species forming following ^63^Cu(I) addition to the ^68^Zn_3_-β MT1A and ^68^Zn_4_-α MT1A domain fragments. The domain fragment emission suggests that Zn_5_Cu_5_-βα MT1A contains a Zn_1_Cu_5_-β cluster and the Zn_4_Cu_6_-βα MT1A, Zn_3_Cu_9_-βα MT1A, and Zn_2_Cu_10_-βα MT1A each contain a Cu_6_-β cluster. The species forming with >10 mol. eq. of ^63^Cu(I) in βα-MT1A exhibit emission from the Cu_6_-β cluster and an α domain cluster. This high emission intensity is seen at the end of the titrations of ^68^Zn_7_-βα MT1A and the ^68^Zn_4_-α MT1A domain fragment suggesting that the initial presence of the Zn(II) results in clustered Cu(I) binding in the α domain.

## Graphical Abstract

**Graphical Abstract:**
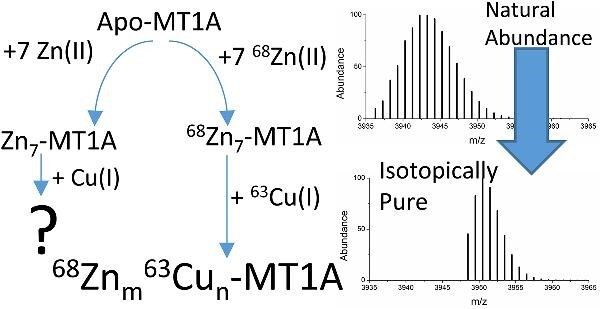
The use of isotopically pure ^63^Cu and ^68^Zn greatly simplifies the ESI-mass spectral data and allows for the unambiguous identification of specific Zn,Cu-MT1A stoichiometries.

## Introduction

Both copper and zinc are essential nutrients that are key to the function of many proteins.^1^ Copper is required in enzymes such as Cu, Zn-dependent superoxide dismutase (SOD) and ceruloplasmin where copper redox chemistry is used to detoxify superoxide radicals^2^ and oxidize iron,^3^ respectively. Zinc is required for the activity of many enzymes and plays a structural role in many additional proteins, for example, zinc finger proteins.^4^ Zinc's Lewis acidity makes zinc an excellent cofactor for enzymes such as carbonic anhydrase and alcohol dehydrogenase.^5^ In total, as many as 3000 human proteins may be zinc proteins.^6^

Metals are tightly regulated in biological systems to avoid unwanted chemistry, such as copper catalysed Fenton reactions.^7,8^ As such, many chaperone and storage proteins with varying affinity constants (K_F_) are involved in metal homeostasis so that metals are shuttled through the cell in a controlled manner.^9^ This results in the concentration of free cellular copper being less than 10^−18^ M.^10,11^ The concentration of free cellular zinc is similarly restricted, with concentrations estimated to be in the picomolar or nanomolar range.^12^

Metallothioneins (MTs) are small molecular weight proteins with a large number of cysteine residues that contribute to homeostatic metal control.^13^ First discovered in equine renal cortex^14^; subsequently, many other MT proteins have been identified through DNA sequence analysis.^15^ MTs are found across multiple branches of life including animals,^16,17^ plants,^17,18^ bacteria,^19^ and fungi.^20^ In mammalian MTs, there are four isoforms and many subisoforms, all containing 20 cysteines. MT1 is predominantly found in the kidneys and MT2 in the liver; however, both can also be found in various amounts throughout the entire body,^21^ whereas MT3 and MT4 are expressed in the central nervous system^22^ and epithelial tissue,^23^ respectively.

**Fig. 1:**
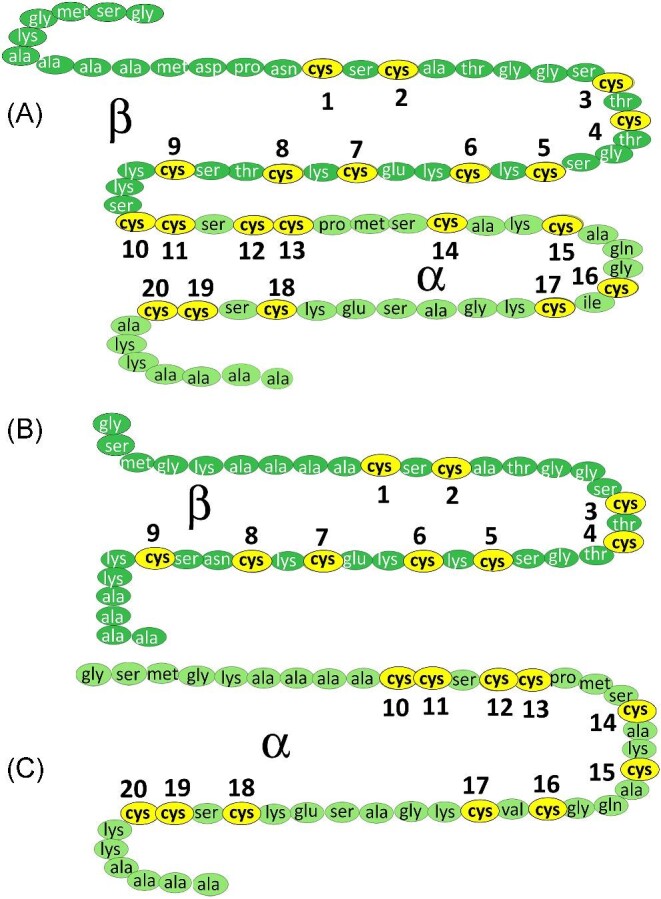
Amino acid sequence for recombinant human metallothionein 1A (A) and its N terminal β (B) and C-terminal α (C) domain fragments. There are 20 cysteines in the full protein identified with yellow ovals numbered 1–20. The numbering of the cysteines in the domain fragments is based on the cysteine numbering in the full protein.

MT proteins lack enzymatic activity, rather the sulphur-containing cysteine residues are used to bind Zn(II) and Cu(I) for metal storage as part of the overall homeostatic pathways and Cd(II) for heavy metal detoxification.^24–34^ MTs may also play a role in redox reactions in the cell.^35,36^ MTs extracted from animal tissues have been reported to contain Zn(II), Cd(II), and Cu(I).^13,37,38^ Many transition metals have also been reported to bind MT *in vitro*.^39–52^  *In vitro*, human MT1 and MT2 can be fully metallated with 7 divalent metals, such as Zn(II) or Cd(II),^53^ or 20 monovalent metals, such as Cu(I).^39^ When mammalian MTs are metallated with exactly 7 divalent metals, two domains form: an N-terminal β domain binding 3 Zn(II) or Cd(II) with 9 cysteines and a C-terminal α domain binding 4 Zn(II) or Cd(II) with 11 cysteines.^53^ The two domains are connected by a short linker region (Fig. 1).^54^  *In vivo*, partially metallated MTs are expected.^55^

Cu-MTs exhibit phosphorescence. The emission spectra recorded, following excitation of the protein at the S-Cu(I) LMCT band (280 nm), have been used to identify the structure of the chromophore as a Cu(I)-thiolate cluster in an environment shielded from the solvent at both 77 K and room temperature.^56–61^ In the absence of Cu(I)-thiolate clusters, very weak emission intensity is expected from isolated Cu(I) bound to the cysteines in MT due to solvent deactivation of the triplet state. This sensitivity of the phosphorescence intensity to the solvent means that the emission is, therefore, sensitive to the overall protein structure. Previously, many researchers reported the emission properties of Cu-MTs frozen at 77 K to show the formation of the Cu-thiolate clusters.^57–59^ However, the value of stepwise metal titrations with MTs has been well established in determining species formation,^62,63^ especially when carried out in parallel with ESI-MS data.^39,60,63^ Metal titrations combined with 77 K emission spectroscopy cannot be carried out without repeated freezing and thawing of the protein. Such constant manipulation of the protein brings the risk of oxidation. Use of a red sensitive spectrofluorometer to measure the room temperature phosphorescence spectra overcomes these difficulties. The use of room temperature emission also allows for relative intensities to be measured which gives useful indications on the overall protein structure based on the relative emissiveness of the clusters formed. The presence of room temperature phosphorescence^39,57,60,61^ further emphasizes that the Cu(I) ions are located in a shielded, clustered environment.

Recently, we have published detailed speciation information on the Cu(I)-thiolate clusters that form in the metal-free apo βα MT1A and its isolated metal-free apo β and α domain fragments.^39,60^ The Cu(I)-thiolate species formed from the apo protein serve as a model for Cu(I) binding to MT newly synthesized by the cell. Cu(I) may also bind to partially Zn-MTs^55^  *in vivo* and so mixed Zn, Cu-MT species are also expected.

A recent paper by Mehlenbacher *et al*. has reported the stoichiometry and structural properties of Cu(I) binding to Zn_7_-MT2 and Zn_7_-MT3.^64^ In that paper, the authors provide detailed stoichiometry and information on Cu(I) binding to the metal-free apo MT2 and MT3 prepared *in situ* using a large excess of glutathione (GSH) to coordinate the initial Zn(II). These authors report that following addition of 20 mol. eq. of Cu(I), both the apo MT2 and apo MT3 bind 8 Cu(I) per protein molecule using inductively coupled plasma mass spectrometric (MS) analysis. Due to the ∼2000 fold excess of GSH, all of the Zn(II) is displaced from the protein upon Cu(I) addition and so Cu(I) and Zn(II) do not coexist in the final protein. It should be noted that *in vivo*, the GSH level is not high enough to displace all of the Zn(II) from MT. The procedure reported by Mehlenbacher *et al*.^64^ is unlike the experimental procedures that we present in this paper where the Zn(II) is retained in the protein as Cu(I) is added stepwise.

Many MT isoforms bind mixtures of Cu(I) and Zn(II) *in vivo* and so the binding pathways for all isoforms are of great interest. MT1 and MT2 must be studied in detail to compare with the more specialized MT3 and MT4 isoforms. MT1 from rat kidneys has been found to contain a substantial amount of Zn(II) and Cu(I).^65^ Zn, Cu-MT1 has been isolated from horse and pig kidneys as well.^66,67^ Zn, Cu-MTs have also been extracted from bovine fetal calf liver with 0–15 Cu(I).^38^ Another well-known example is the Zn, Cu-MT3 isolated from bovine brain with 4–5 Cu(I) and 2–2.5 Zn(II) ions ^68^ and from human brains with 4 Cu(I) ions and 3 Zn(II) ions.^22^ The metallation of MTs may also change with disease. Human hepatocellular carcinoma cells have been reported to contain, Zn, Cu-MT, and Cu-MT.^69^ A significant complication when assessing the metallation status of MTs formed *in vivo* is that the natural balance between Zn(II) and Cu(I) may be disrupted by the extraction procedures of these very air-sensitive proteins. Therefore, stepwise additions of Cu(I) into dilute solutions of Zn-saturated or partially saturated Zn-MT in controlled oxygen-free environments are of particular importance in establishing potential *in vivo* speciation.

Many of the techniques used in previous studies of Zn, Cu containing MTs only report on the average metal content. However electrospray ionization (ESI) MS studies have shown that mixtures of species should be expected.^39,60,70^ The presence of multiple species forming with varying degrees of cooperativity means that the mol. eq. of copper added is not always a reliable technique for determining the exact stoichiometry of the species formed. This may explain the variation in reported Zn, Cu-MT stoichiometries obtained from optical studies. Examples include Stillman *et al*. who reported the formation of Zn_4_Cu_6_, Cu_12_, and Cu_20_ species from rabbit liver Zn_7_-MT2 using circular dichroism spectroscopy and room temperature emission spectroscopy methods,^71^ Bogumil *et al*. who reported a Cu_4_ cluster within Cu, Zn-MT3 using 77 K emission spectroscopy,^68^ and Bofill *et al*. who concluded from UV-visible absorption and circular dichroism spectroscopy that 4 Cu(I) bind cooperatively to the isolated β domain fragment of mouse MT1. In that latter study, Chelex-100 was added to bind the Zn(II) displaced by the incoming Cu(I); analysis revealed that 2 mol. equiv. of Zn(II) were bound to the Chelex-100 leading the researchers to conclude that a Zn_1_Cu_4_ β mouse MT species formed. However, in none of these previously mentioned studies ESI-MS was used to confirm the speciation.^68,71,72^

A later study by Bofill *et al*. reported several Zn, Cu-MT species forming in mouse MT1 using ESI-MS methods.^73^ In their paper, the masses listed for each species decreased over the course of the titration, suggesting that there may be additional Zn, Cu species forming that the authors could not resolve. An example of the difficulty in resolving mixed Zn, Cu species is also seen in the following studies on Zn, Cu speciation in snail MT. In these papers, the authors report only the total number of metals bound (M_x_, where x is the sum of both Zn(II) and Cu(I) ions bound).^74,75^ The issue in distinguishing Zn(II) from Cu(I) lies not only in the similar averages masses but in the overlapping isotopic distributions of Cu(I) and Zn(II). While high resolution mass spectrometers are able to distinguish between very small mass differences, the broad MS peaks that arise due to using natural abundance Zn(II) and Cu(I), in addition to the isotopic distribution from the C, H, N, O, and S in the protein, make it very difficult to clearly determine the Zn/Cu exchange process that takes place as Cu(I) is added to Zn-MTs. We note that even with the 140 000 resolution of the Q-Exactive^TM^ Orbitrap mass spectrometer (data unpublished), it was impossible to clearly identify the exact stoichiometries of the Zn, Cu-MT species of similar masses with >3 Cu(I) and Zn(II).

In the studies described in this present paper, we overcome these significant and longstanding difficulties and report the unambiguous stoichiometry of a series of Zn, Cu-βα MT1A species at pH 7.4 through the use of isotopically pure ^68^Zn(II) and ^63^Cu(I) when Cu(I) is added to human Zn_7_-βα MT1A. Our results show that the Zn(II) is retained in the protein with up to 14 Cu(I). Through the use of the domain fragments, we identify the localization of the remaining Zn(II). Room temperature solution emission spectra measured in tandem with the stepwise ESI-MS data provide evidence for the presence of a series of distinct Zn, Cu(I)-thiolate clustered species. The data show that the specific species that form with an increasing number of metal ions are controlled by the total positive charge from the sum of the Zn(II) and Cu(I) ions. For displacement of Zn(II) by Cu(I), this means that the oxidation state of the incoming metal ion (here + 1) is an important criterion in terms of the specific stoichiometric ratios, Zn: Cu, that are stable at equilibrium.

## Methods

### MT synthesis

βα MT1A and its N-terminal β domain fragment and C-terminal α domain fragment were made recombinantly using BL21(DE3) *Escherichia coli* cells containing the MT1A gene in a pET29a vector as published previously.^60^ The recombinant human MT1A protein had the sequence MGKAAAAMDP NCSCATGGSC TCTGSCKCKE CKCTSCKKSC CSCCPMSCAK CAQGCICKGA SEKCSCCAKK AAAA. The N-terminal β domain fragment, referred to as β MT, had the sequence MGKAAAACSC ATGGSCTCTG SCKCKECKCN SCKKAAAA. The C-terminal α domain fragment, referred to as α MT, had the sequence MGKAAAACCS CCPMSCAKCA QGCVCKGASE KCSCCKKAAA A. An N-terminal S-tag with the sequence MKETAAAKFE RQHMDSPDLG TLVPR GS was used to increase protein stability *in vivo*. The tag was cleaved off before any metallation experiments. Tag cleavage resulted in a ‘GS’ left on the N-terminal of the protein. We note that the extra N- and C-terminal flanking sequences have insignificant effect on the metal binding properties of MT.

Cells from a glycerol stock were incubated in autoclaved LB broth (Millipore Sigma) overnight and added to additional LB broth the following morning. The addition of cadmium acetate (Millipore Sigma) during *E. coli* growth ensured that all MT produced was saturated with cadmium. Isopropyl β-d-1-thiogalactopyranoside (IPTG) (BioShop Canada) was added once the A_600_ reached 0.6. The cells were harvested by centrifugation at 8983 × *g* 4 h after the addition of IPTG and resuspended in pH 7.4 *tris* (hydroxymethyl)aminomethane (Tris) buffer (Millipore Sigma). The cell suspension was stored at –80°C until protein purification.

During the purification process, cells were lysed using a cell disruption system (Constant Systems, UK) at 18, 20, and 22 kpsi. All buffers used in the purification process were saturated with argon to prevent protein oxidation. The cell lysate was centrifuged at 20 442 × *g* and the lysate was loaded onto two 5 ml HiTrap SP Sepharose cation exchange columns (Cytiva). Protein elution was monitored by the 250 nm S-Cd ligand to metal charge transfer band (LMCT) measured by a Cary 50 UV-Vis Spectrophotometer (Varian). The collected protein was concentrated to a final volume of 20 ml using a stirred ultrafiltration cell (EMD Millipore) with a 3 kDa cellulose membrane under nitrogen pressure. The Thrombin CleanCleave kit (Millipore Sigma) was used to remove the S-tag from the MT. Thrombin resin was removed from the protein sample by centrifugation, and the purified protein was separated from the S-tag fragment using the HiTrap SP Sepharose cation exchange columns again. Purified protein was stored at –20°C.

### Formation of ^68^Zn-MT

All solutions were thoroughly evacuated and saturated with argon before use. Cadmium saturated MT was demetallated and desalted using a PD10 column (Cytiva) containing pH 2.5 ammonium formate. Excess Cd(II) was removed by buffer exchange with argon-saturated ammonium formate (J. T. Baker) using 3 kDa centrifugal filters (Amicon). The pH of the protein was raised with an additional PD10 column containing argon-saturated pH 7.4 ammonium formate. Tris(2-carboxyethyl)phosphine (TCEP) was included in all ammonium formate solutions to prevent thiol oxidation. The protein concentration was measured by metallating a fraction of the protein sample with cadmium and measuring the absorbance at 250 nm (S-Cd LMCT). ^68^ZnO was purchased from Trace Sciences International and dissolved in dilute acetic acid with heating. The pH was increased to 4.1 with dilute NH_4_OH and diluted to a final concentration of 10 mM with Milli Q water. The ^68^ Zn solution was deaerated and argon-saturated before being titrated into the apo protein until the majority of the protein was ^68^Zn_7_-βα MT1A, ^68^Zn_3-_β-MT1A, or ^68^Zn_4_-α MT1A as seen by the ESI-MS. The pH of the ^68^Zn-MT1A was adjusted to 7.4.

### Cu(I) titrations

Isotopically pure ^63^CuCl_2_ was obtained from Trace Sciences International. Reduced GSH (Sigma) was dissolved in argon-saturated 10 mM ammonium formate at pH 7.4. ^63^CuCl_2_ was added at a 3:1 GSH: ^63^Cu(II) ratio to form reduced Cu(I)-GSH as reported by Ferreira *et al*.^76^ All ^63^Cu(I) solutions were made to be 10 mM. All solutions were rigorously deaerated and argon-saturated to prevent oxidation. Molar equivalents of the 10 mM ^63^Cu-GSH were added stepwise (based on the MT concentration) to the Zn-saturated protein assuming all Cu(II) was reduced to Cu(I). For each addition of ‘Cu(I)’, we mean the addition of Cu(I) bound to an unknown number of GSH ligands and the oxidized GSSG product. The Cu(I)(SG)_x_ species are not observed in the ESI-MS so the value of x cannot be determined at this time by our methods. All Cu(I) was added to protein at pH 7.4 and room temperature. Zn, Cu-MT samples were measured either by ESI-MS or emission spectroscopy approximately 30 s after each addition as there were insignificant changes in the mass spectrum or the emission spectrum after this time point. There was no indication of oxidation of the protein that would result from Cu(I) disproportionation or any evidence of coloured, oxidized Cu(II).

### Electrospray ionization mass spectrometry

Samples prepared by the methodology above were measured by direct infusion into the Bruker MicrOTOF II (Bruker Daltonics) in positive ion mode. The parameters used are shown in Table 1. All samples were rigorously deaerated and argon-saturated to prevent oxidation. The relative abundance of each species detected was normalized and the mol. eq. of Cu(I) bound to the protein was calculated from those values.

**Table 1.** ESI-MS parameters

Parameter	Value
End plate offset	500 V
Capillary voltage	4500 V
Nebulizer	29.0 psi
Dry gas	6.0 L/min
Dry temp	200°C
Target mass range	800–3000 m/z
Capillary exit voltage	180.0 V
Hexapole RF	600 Vpp

### Emission spectroscopy

Room temperature Cu(I)-thiolate cluster phosphorescence was measured using a Photon Technology Quanta Master 4 (QM4) scanning spectrofluorometer (PTI Inc., London, Canada). Protein samples (prepared by the methodology above) were kept anaerobically in a 1 cm sealed quartz cuvette to prevent oxidation. Samples were excited at 280 nm using a Xenon flash lamp (flash rate = 100 Hz), and the resulting room temperature phosphorescence was measured from 500–900 nm with a 750 nm blazed grating and a red-sensitive GaAs phototube. A yellow filter was used to decrease the scatter of the excitation overtone band. All slits were held constant at 10 nm. The Xenon flash lamp allowed for phosphorescent lifetime measurements. Since different Zn, Cu-MT species have different wavelengths of emission, the presence of mixtures in the solution results in multiple overlapping band envelopes with the λ_max_ representing an average. The species contributing to the emission were identified by measuring the ESI-MS profiles for each point in the titration alongside the emission measurements.

The phosphorescent decay of selected wavelengths was measured after exciting the sample at 280 nm. The emission was measured for 20–30 μs in 100 steps with a 1–2 μs integration time. When multiple emission bands are present, wavelengths on the edge of the emission band envelope away from other emission bands were selected to decrease the chance of measuring an average lifetime of the two emission bands. The lifetimes were calculated using the QM-4 software. All samples were rigorously deaerated and argon-saturated to prevent oxidation.

### Mass spectrometry isotope simulations

Simulated MS data were generated using Compass IsotopePattern (Bruker) with the resolution set to 10 000. The isotope pattern for apo-β MT1A was calculated by inputting the protein sequence. For the simulations of the metallated β MT1A species, 2 protons were removed for each Zn(II) bound and 1 proton for each Cu(I) bound. In a deconvolution calculation, the charge of the metal ions is compensated for the loss of the same number of protons, independent of the apparent coordination numbers. For example, the 14+ charge of 7 Zn(II) is compensated for in the calculation by the loss of 14 protons. These numbers are observed in the experimental ESI-MS data upon metal binding. The program calculated the abundance of each isotopomer with different combinations of carbon, hydrogen, nitrogen, oxygen, and sulfur isotopes as well as the zinc and copper, when natural abundances were being considered. Isotopically pure ^68^Zn and ^63^Cu were added to the simulated mass spectra manually as the Compass Isotope Pattern program does not have a function for simulating isotopically pure elements. The simulations of apo-β MT1A, Zn_3_-β MT1A, Zn_1_Cu_5_-β MT1A, and Cu_6_-β MT1A were compared to the experimental data. The contributions of Zn_1_Cu_5_-β MT1A and Cu_6_-β MT1A to the experimental M_6_ peak were determined by adding together varying fractions of the isotope pattern for isotopically pure ^63^Cu_6_-β MT1A and ^68^Zn_1_^63^Cu_5_-β MT1A until the simulation matched the experimental data. The contributions of ^68^Zn_2_  ^63^Cu_4_-α MT1A and ^68^Zn_3_  ^63^Cu_3_-α MT1A to the experimental M_6_ peak were determined by adding together varying fractions of the isotope pattern for isotopically pure ^68^Zn_2_  ^63^Cu_4_-α MT1A and ^68^Zn_3_  ^63^Cu_3_-α MT1A until the simulation matched the experimental data. The contributions of Zn_5_Cu_5_-βα MT1A and Zn_4_Cu_6_-βα MT1A to the experimental M_10_ peak were determined by adding together varying fractions of the isotope pattern for isotopically pure ^68^Zn_4_^63^Cu_6_-β MT1A and ^68^Zn_5_^63^Cu_5_-β MT1A until the simulation matched the experimental data.

## Results and discussion

### Isotope simulations prove the necessity of using ^63^Cu(I) and ^68^Zn(II) in mixed metal binding to MTs

MS is key to determining the exact stoichiometry of the many metallated species of MT. Previously, researchers have had considerable difficulty in determining accurate Zn, Cu-MT stoichiometries.^74,75^ Even with high-resolution mass spectrometry (Q-Exactive^TM^ Orbitrap mass spectrometer) (data unpublished), we were still unable to determine the exact stoichiometry without ambiguity even with a higher resolution of 140 000.

**Fig. 2:**
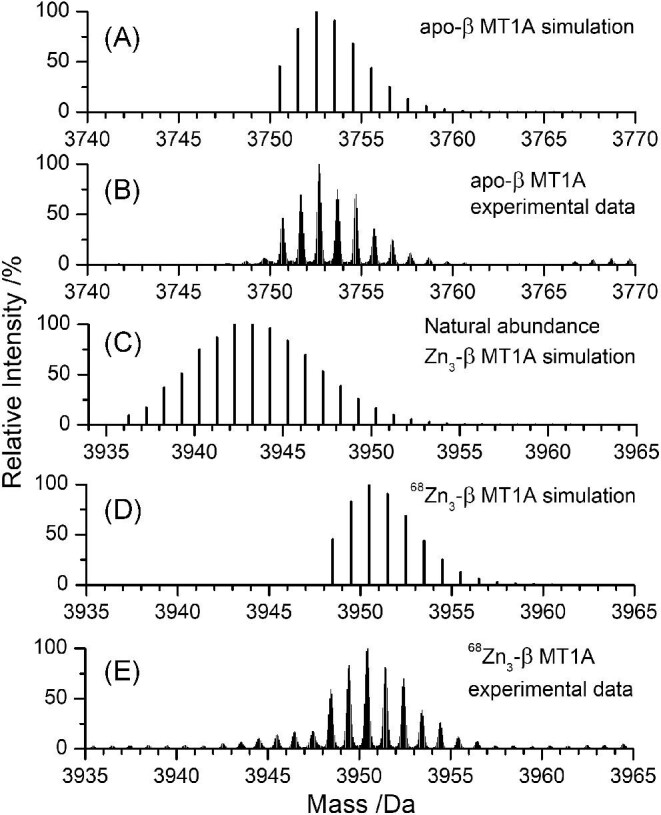
Simulated and experimental mass spectral data showing the isotopic distributions of apo-β MT1A (A, B) and Zn_3_-β MT1A (C–E). (A) Simulated isotopic pattern of apo-β MT1A. (B) Experimental ESI-mass spectral data of apo-β MT1A. (C) Simulated isotopic pattern for natural abundance Zn_3_-β MT1A. (D) Simulated isotopic pattern for isotopically pure ^68^Zn_3_-β MT1A. (E) Experimental ESI-mass spectral data of ^68^Zn_3_-β MT1A. Note the x-axis ranges differ between A, B and C, D, E because of the additional mass of the three Zn(II) ions bound.

**Fig. 3:**
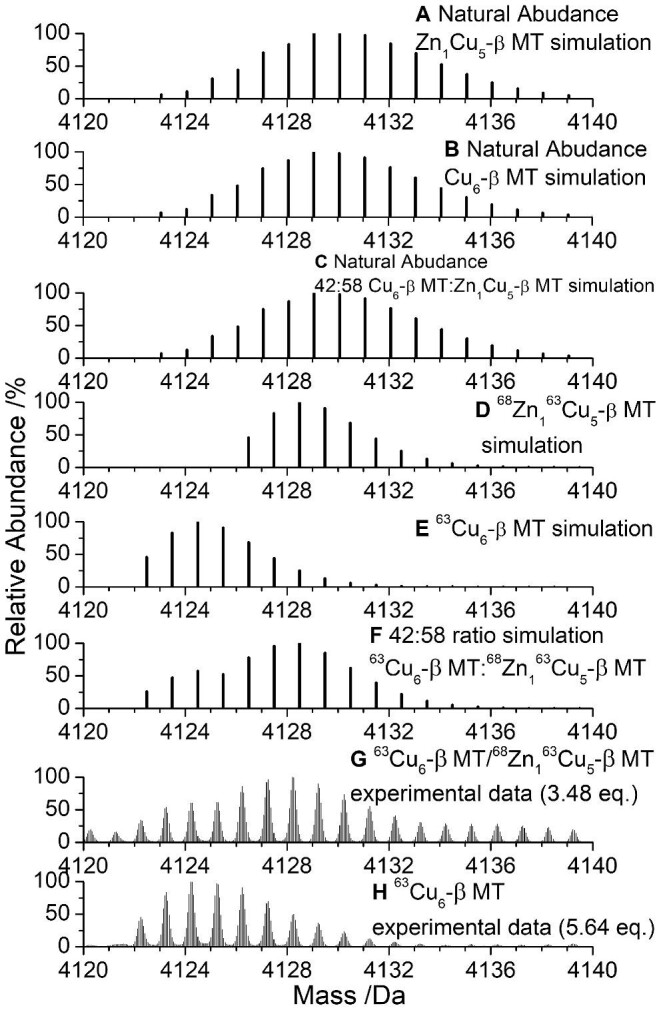
Simulated and experimental mass spectral data showing the isotope patterns for Zn_1_Cu_5_-β MT1A and Cu_6_-β MT1A. (A) Simulated isotope pattern for natural abundance Zn_1_Cu_5_- β MT1A. (B) Simulated isotope pattern for natural abundance Cu_6_-β MT1A. (C) Simulated isotope pattern for a 42:58 ratio of natural abundance Cu_6_-β MT1A: Zn_1_Cu_5_- β MT1A. (D) Simulated isotope pattern for isotopically pure ^68^Zn_1_^63^Cu_5_-β MT1A. (E) Simulated isotope pattern for isotopically pure ^63^Cu_6_-β MT1A. (F) Simulated isotope pattern for a 42:58 ratio of isotopically pure ^63^Cu_6_-β MT1A:^68^Zn_1_^63^Cu_5_-β MT1A. (G) Experimental isotope pattern for a mixture of isotopically pure ^63^Cu_6_-β MT1A and ^68^Zn_1_^63^Cu_5_-β MT1A formed from binding 3.48 mol. eq. of ^63^Cu(I) to 52.8 μM Zn_3_-β MT1A. (H) Experimental isotope pattern for ^63^Cu_6_-β MT1A formed from binding 5.64 mol. eq. of ^63^Cu(I) to 52.8 μM Zn_3_-β MT1A. The Cu(I) equivalences for (G) and (H) were determined through analysis of the ESI-mass spectral data.

In Figs 2 and 3 (and later 6, 8, and 12), we use the program, ‘Compass IsotopePattern’, to simulate mass spectra and demonstrate that the problem is the multiple, overlapping stable Zn(II) and Cu(I) isotopes (Table 2) that combine with the broad peptide MS envelope. The protein peak is broad due to the many combinations of carbon isotopes, particularly ^12^C and ^13^C, as well as isotopes of H, N, O, and S isotopes in the protein. The effects of the broad protein peak and the multiple Zn(II) and Cu(I) isotopes make it impossible to distinguish between the mass of Zn(II) and Cu(I). The use of isotopically pure metals dramatically improves species identification without the need for more specialized high-resolution mass spectrometers. The use of isotopically pure elements has also been used to study metal exchange^77,78^ as well as the formation of other metal-sulfur clusters.^79^ We note that these simulations use the β domain fragment and that the complexity is further exacerbated when the full βα-MT1A protein is metallated.

**Table 2.** Stable isotopes of copper and zinc

Isotopes of Cu	Abundance
^63^Cu	69.15%
^65^Cu	30.85%
**Isotopes of Zn**	**Abundance**
^64^Zn	49.17%
^66^Zn	27.73%
^67^Zn	4.04%
^68^Zn	18.45%
^70^Zn	0.61%

### The clarity in the measurement of Zn_3_-β MT using isotopically pure ^68^Zn(II)

To confirm the validity of the simulations, the isotope pattern of the apo protein (Fig. 2A) was first modelled and compared to experimental ESI-MS data (Fig. 2B). There is good agreement between the experimental and simulated mass spectra. Metallated β MT1A species were then simulated. The isotopic pattern for natural abundance Zn_3_-β MT1A becomes very complicated with 21 isotopomers as a result of the 5 stable Zn(II) isotopes (Fig. 2C). The result is that the peak, centred on 3943 Da, is very broad (Fig. 2C).

In contrast, the width of the simulated isotope pattern for the isotopically pure ^68^Zn_3_-β MT1A (Fig. 2D) is identical to that of the apo protein as expected for the addition of an element with a single isotope. The experimental isotope pattern of ^68^Zn_3_-β MT1A is shown in Fig. 2E. The experimental band envelope is an almost identical copy of the simulation (Fig. 2D), except with some additional low-intensity peaks corresponding to ^68^Zn_2_^63^Cu_1_-β MT1A centred on 3946 Da. This species formed as a result of residual ^63^Cu(I) in the ESI-MS PEEK tubing. It is the use of isotopic ^68^Zn(II) and ^63^Cu(I) that allows for the detection of the adjacent ^68^Zn_2_^63^Cu_1_-β MT1A species. If natural abundance Zn(II) were used, this species would be indistinguishable from the main Zn_3_-β MT1A peak.

### Use of isotopically pure ^63^Cu(I) and ^68^Zn(II) greatly increases clarity in the ESI-MS of isotopically pure ^68^ Zn,^63^Cu-β MT1A species

While Cu(I) only has two stable isotopes, the isotopic pattern quickly becomes complicated when multiple Cu(I) ions are considered. The combination of the broad protein MS peak and the overlapping Zn(II) and Cu(I) isotopes essentially makes it impossible to distinguish species with the same total number of metals but differing Zn: Cu ratios. This is seen in the simulations of the Zn_1_Cu_5_-β MT1A (Fig. 3A) and Cu_6_-β MT1A (Fig. 3B) MS peaks as well as the simulation of the 42:58 ratio of Cu_6_-β MT1A: Zn_1_Cu_5_-β MT1A. It is, therefore, not surprising that previous reports of Cu and Zn stoichiometries in Zn, Cu MT species using ESI-MS and natural abundance Zn(II) and Cu(I) have been unable to resolve the presence of specific species.^74,75^

The isotopic pattern is again compressed with the use of the isotopically pure metals. The reduction in the breadth of the peak as well as the greater mass difference between ^63^Cu(I) and ^68^Zn(II) (compared to the average natural abundance masses) allows for the clear distinction between ^68^Zn_1_^63^Cu_5_-β MT1A (Fig. 3D, 4128.5 Da) and ^63^Cu_6_-β MT1A (Fig. 3E, 4124.5 Da).

In the case where ^68^Zn_1_^63^Cu_5_-β MT1A and ^63^Cu_6_-β MT1A exist at the same point in the titration, we can simulate the overall MS envelope for any given ratio of the two species (*vide infra* Fig. 6). A ratio of 42:58 ^63^Cu_6_-β MT1A:^68^Zn_1_^63^Cu_5_-β MT1A (Fig. 3F) best matched the experimental data in Fig. 3G. The experimental ESI-MS peak for ^63^Cu_6_-β MT1A is centred on 4124.5 Da (Fig. 3H), similar to the simulated ^63^Cu_6_-β MT1A mass spectrum (Fig. 3E).

Supplementary Figs S1 and S2 show the experimental results of adding natural abundance Cu(I) to ^68^Zn_3_-β MT1A. It is clear from Supplementary Fig. S2 that using both isotopically pure ^63^Cu(I) and ^68^Zn(II) results in much better separation between Zn_1_Cu_5_-β MT1A and Cu_6_-β MT1A; however, in the absence of isotopically pure Cu(I), room temperature emission and circular dichroism spectra (Supplementary Fig. S1) aid in the identification of two separate species.

### Use of ^63^Cu(I) and ^68^Zn(II) reveals a series of mixed-metal Zn, Cu-MT clustered species

**Fig. 4:**
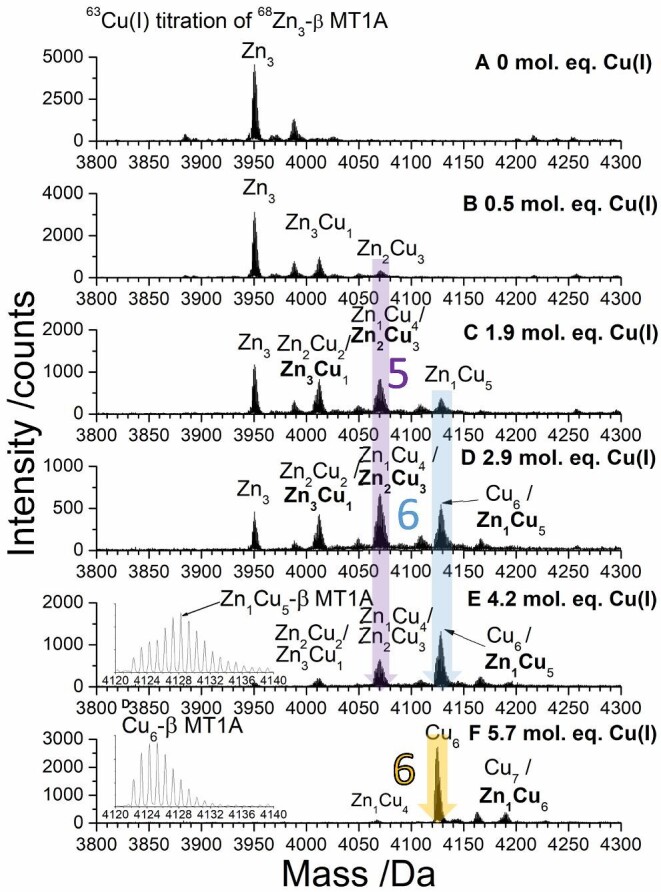
Deconvoluted ESI-mass spectral data showing key steps in the ^63^Cu(I) titration of 29.6 μM ^68^Zn_3_-β MT1A titration. All steps in the titration are shown in Supplementary Fig. S3. Charge state data are shown in Supplementary Fig. S4. 3D speciation data are shown in Supplementary Fig. S5. The masses of all species detected are in Supplementary Table S1. The mol. eq. refer to the mol. eq. of Cu(I) bound to the protein as determined by ESI-MS.

Using isotopically pure ^68^Zn(II) acetate and ^63^Cu(I)-GSH, our detailed ESI-MS results discussed below show the Zn(II)/Cu(I) exchange reactions first in the Zn_3_-β MT1A (Fig. 4) and Zn_4_-α MT1A (Fig. 7) domain fragments. These results are used to explain the process of Zn(II)/Cu(I) exchange in the Zn_7_-βα MT1A (Fig. 10). The masses of all the species detected by ESI-MS are listed in Supplementary Tables S1–S3. While Zn_7_-MT1A may not occur in the cell, these experiments serve as a basis for future studies of partially metallated MTs using this method. In the subsequent descriptions, we omit the isotopically pure descriptor for the Cu(I) and Zn(II) and the GSH ligand for the Cu(I). The displaced Zn(II) can be bound by the GSH; however, the binding constants for the Zn(II) and Cu(I) in the various clustered structures of MT are much greater^39,80^ than the binding constants for Cu(I) or Zn(II) binding to the GSH^11,64,81^ so the presence of GSH does not significantly change the outcome of the titration of Cu(I) into Zn-MT.

### Cu(I) metallation of ^68^Zn_3_-β MT1A results in the formation of Zn_1_Cu_5_-β MT1A, then Cu_6_-β MT1A

**Fig. 5:**
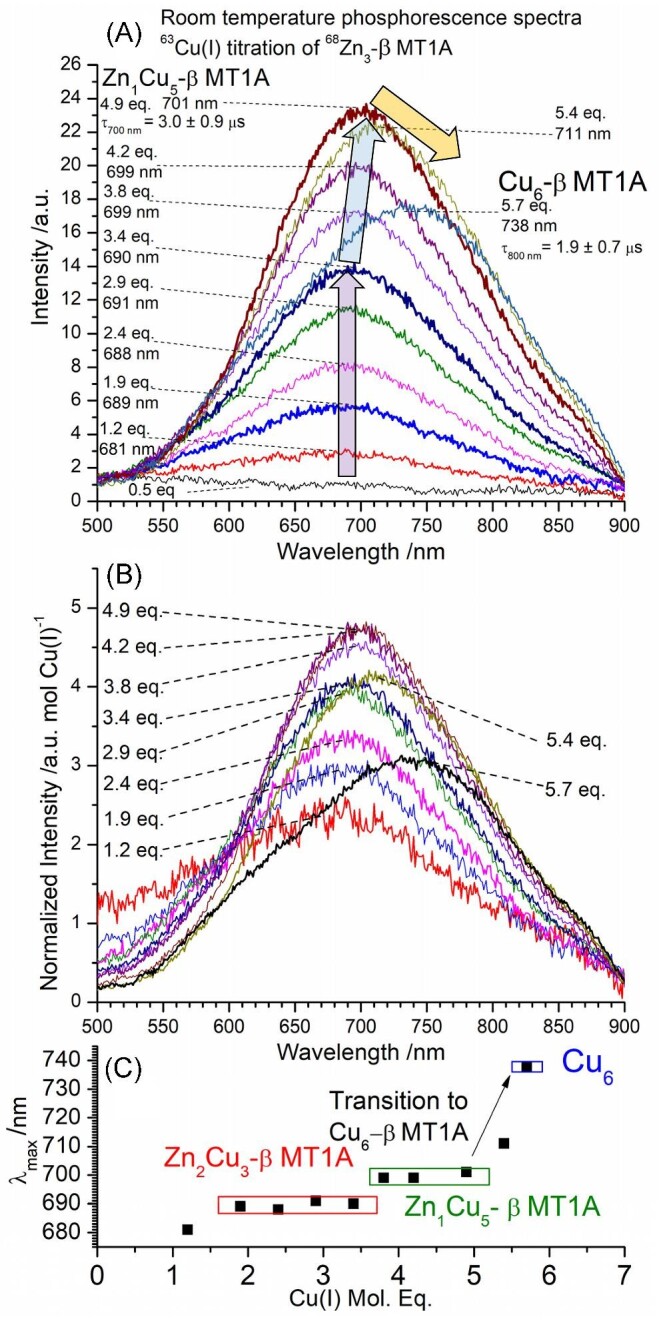
Room temperature phosphorescence spectra recorded during a ^63^Cu(I) titration of ^68^Zn_3_-β MT1A. (A) Emission spectra showing relative intensity changes throughout the titration. λ_ex_ = 280 nm. (B) Emission spectra normalized for the amount of Cu(I) bound at each step in the titration. (C) λ_max_ as a function of Cu(I) bound to the protein. Phosphorescence decay traces are shown in Supplementary Fig. S6. The mol. eq. refer to the mol. eq. of Cu(I) bound to the protein as determined by ESI-MS.

To gain insight into the pathways involved when Cu(I) replaces Zn(II) in the β domain, ^68^Zn(II) was added to the β domain fragment to make ^68^Zn_3_-β MT1A at pH 7.4 and room temperature. The resulting species, formed after 11 stepwise additions of Cu(I), were measured by ESI-MS (Fig. 4) and room temperature emission spectroscopy (Fig. 5). The approach of connecting the emission data with parallel measurement of the speciation using ESI-MS allows for the unambiguous identification of the key Zn, Cu-MT species contributing to the phosphorescence spectra. The ESI-MS data clearly show that at certain steps multiple species are present resulting in an average emission band envelope comprising one or more specific bands. Because isotopic ^68^Zn(II) and ^63^Cu(I) were used, the stoichiometry is well known and specific band centres can be discerned from the overall spectral envelope allowing the identity of the contributing species with increased certainty.

Significant changes in the ESI-MS data are highlighted with coloured arrows and numbers indicating the total number of metals bound. The changes in the emission spectra generated by these species are highlighted using the same coloured arrows. In the cases where two species result in adjacent peaks in the mass spectrum (e.g., Zn_1_Cu_5_-β MT1A and Cu_6_-β MT1A), the two species are separated by a slash and the major species is bolded. While these are not visible as two separate peaks when looking at the entire mass region, zoomed in regions of key peaks have been added as insets. Due to the complexity of the mixtures of Zn, Cu-MT species, we describe the data in terms of the change in the number of metal ions bound.

*M_3_ to M_4_/M_5_*. The first addition of Cu(I) to Zn_3_-β MT1A results in the formation of an M_4_ species, Zn_3_Cu_1_-β MT1A, and a smaller amount of M_5_ species, Zn_2_Cu_3_-β MT1A (Fig. 4B, Zn_2_Cu_3_ labelled with purple arrow and ‘5’ for the total metals bound). Additional Cu(I) results in an increase in the abundance of both of these species and one Zn(II) in a fraction of both species is replaced by Cu(I) to form minor amounts of Zn_2_Cu_2_-β MT1A and Zn_1_Cu_4_-β MT1A (Fig. 4C).

*M_4_/M_5_ to M_6_*. The next species to form is M_6_ which forms initially as Zn_1_Cu_5_-β MT1A (light blue arrow, blue ‘6’). We note that the formation of both Zn_2_Cu_3_-β MT1A and Zn_1_Cu_5_-β MT1A is preferred over the M_4_ species, as evident by the formation of these species before Zn_3_-β MT1A is fully consumed. The formation of the Zn_2_Cu_3_-β MT1A is associated with emission intensity (Fig. 5A) at 690 nm (purple arrow). As the abundance of Zn_1_Cu_5_-β MT1A increases in the MS data, the emission grows in intensity and redshifts to a λ_max_ = 701 nm with 4.9 mol. eq. Cu(I) bound (blue arrow).

**Fig. 6:**
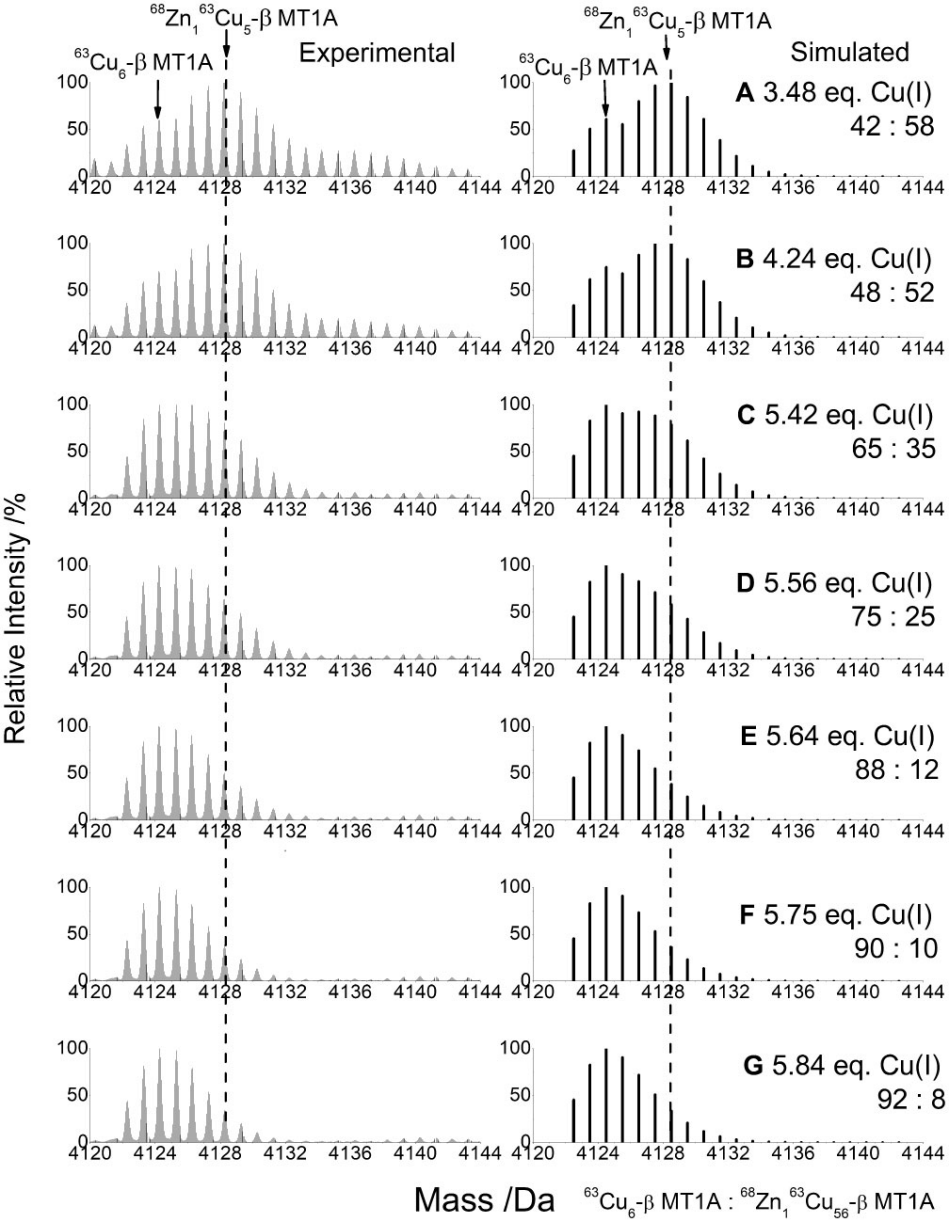
Series of experimental and simulated mass spectra showing the transition from ^68^Zn_1_^63^Cu_5_-β MT1A to ^63^Cu_6_-β MT1A formed during the ^63^Cu(I) titration of 29.6 μM ^68^Zn_3_-β MT1A titration. Full titration is shown in Fig. 4. The dashed line indicates the middle of the initial peak.

Additional Cu(I) results in a gradual change from Zn_1_Cu_5_-β MT1A (blue arrow) to Cu_6_-β MT1A in the ESI-MS (yellow arrow), until the Cu_6_-β MT1A becomes the major species at 5.7 mol. eq. Cu(I) bound. The insets in Fig. 4E and F show clear differences between the Zn_1_Cu_5_-β MT1A and Cu_6_-β MT1A when the mass region is expanded to show only the M_6_ mass region. The change is clearer in Fig. 6, where the M_6_ peak is shown for seven steps in the titration, ranging from 3.48 mol. eq.to 5.84 mol. eq. bound. The experimental data was compared to MS simulations to determine the ratio of Cu_6_-β MT1A: Zn_1_Cu_5_-β MT1A. The transition from these two species is accompanied by a redshift in the Zn_1_Cu_5_-β MT1A emission spectrum with λ_max_ = 701 nm (4.9 mol. eq. Cu(I)) to the Cu_6_-β MT1A emission spectrum with λ_max_ = 737 nm (5.7 mol. eq. Cu(I)). The Zn(II), with its typically tetrahedral coordination in MT, is likely located within the cluster to share the thiols with some of the Cu(I) ions. The nine cysteines of the β domain can bridge the one Zn(II) and 5 Cu(I) ions. We note that when Cu(I) is added to the apo-β MT1A, a Cu_7_S_9_ cluster is a dominant structure with a unique emission spectrum^60^ which suggests the replacement of two Cu(I) with one Zn(II) while retaining the cluster structure is reasonable. The emission intensity also drastically drops at this point, indicating that a different structure has formed.

The drop in emission intensity is especially evident in Fig. 5B, where the emission intensity has been normalized for the mol. eq. of Cu(I) bound. The lower emission intensity suggests that the Cu_6_-β MT1A cluster and/or the protein backbone wrapped around it is more open or porous than the Zn_1_Cu_5_-β MT1A cluster, which leads to an increase in triplet state deactivation. This is also seen directly in the luminescence decay profiles with Zn_1_Cu_5_-β MT1A having a lifetime of 3.0 ± 0.9 μs (λ_em_ = 700 nm) and Cu_6_-β MT1A having a lifetime of 1.9 ± 0.7 μs (λ_em_ = 800 nm) (Supplementary Fig. S6).

Figure 5C summarizes the emission of the key species by showing the λ_max_ as a function of Cu(I) bound to the protein. With the first 2–4 mol. eq. of Cu(I) bound, the λ_max_ stays constant at about 690 nm and is characteristic of the Zn_2_Cu_3_-β MT1A species. From 4–5 mol. eq. Cu(I) bound, there is a shift in the λ_max_ to approximately 700 nm, characteristic of the Zn_1_Cu_5_-β MT1A species. From 5–6 mol. eq. Cu(I) bound, there is a further shift in the λ_max_ to approximately 740 nm which is characteristic of the Cu_6_-β MT1A species. A similar emission spectrum is seen with the formation of Cu_6_ from the apo domain fragment.^60^

*M_6_ to M_7_*. Only a small amount of an M_7_ species, Zn_1_Cu_6_-β MT1A, forms even with excess Cu(I) added (Fig. 4F). To test whether the Cu_6_-β MT1A was kinetically inhibited from expanding to Cu_7_-β MT1A, the sample containing Cu_6_-β MT1A formed from Zn_3_-β MT1A was heated from room temperature to 60°C and allowed to equilibrate for 2 min before the mass spectrum was measured again (Supplementary Fig. S7). Despite heating, no further metallation of the Cu_6_-β MT1A was observed. This is unlike the Cu(I) titration into the apo-β MT1A domain fragment where all of the protein is readily converted from Cu_6_-β MT1A to Cu_7_-β MT1A.^60^ This suggests that the Cu_6_-β MT1A species that forms from Zn_3_-β MT1A is a different structure and that significant rearrangement would likely be necessary to bind a 7^th^ Cu(I) to the protein. It is clear that the M_6_ species in the β domain are preferentially formed over species with fewer metals, whether they are comprised only Cu(I) or a mixture of Zn(II) and Cu(I).

### ^63^Cu(I) metallation of ^68^Zn_4_-α MT1A results in a continuum of mixed Zn, Cu MT species

**Fig. 7:**
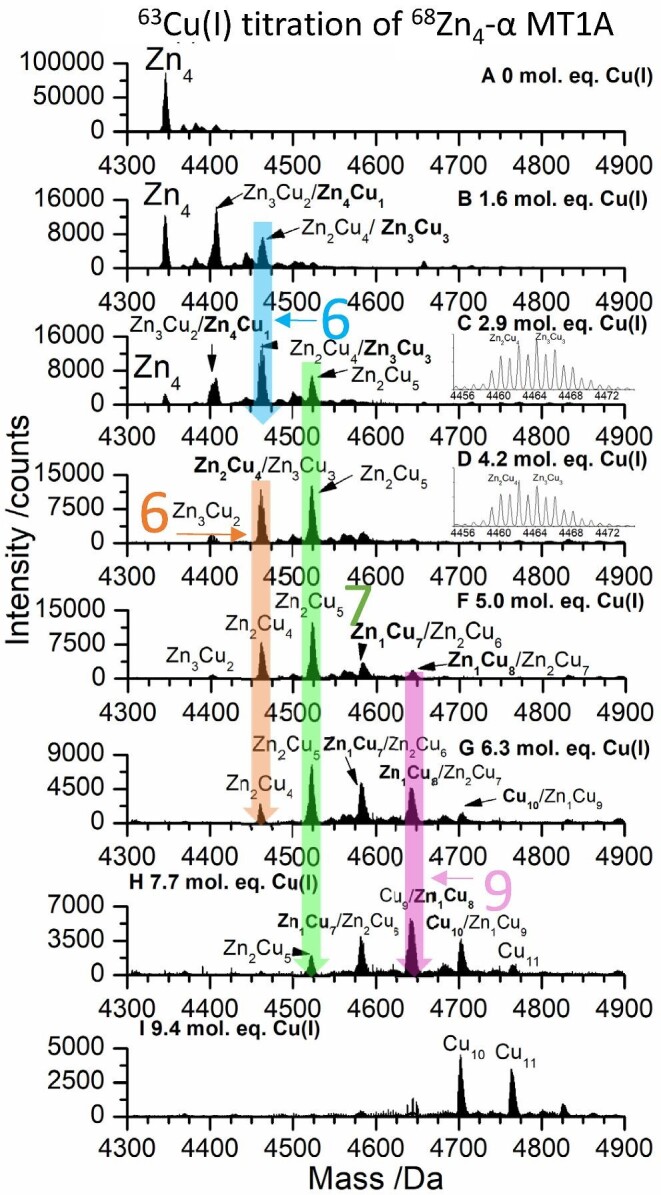
Deconvoluted ESI-mass spectral data showing key steps during the ^63^Cu(I) titration of 35 μM ^68^Zn_4_-α MT1A at pH 7.4 and room temperature. All steps of the titration are shown in Supplementary Fig. S8. Charge state data are shown in Supplementary Fig. S9. 3D speciation data are shown in Supplementary Fig. S10. The masses of all species detected are listed in Supplementary Table S2. The mol. eq. refer to the mol. eq. of Cu(I) bound to the protein as determined by ESI-MS.

To investigate the Cu(I) replacement of the four Zn(II) ions in the α domain of MT1A, ^63^Cu(I) was titrated into ^68^Zn_4_-α MT1A. The resulting species were measured by both ESI-MS (Fig. 7) and emission spectroscopy (Fig. 9). The coloured arrows highlight key species in the mass spectra and show the corresponding changes in the emission spectra. In the case where two species result in adjacent peaks in the mass spectrum (e.g., Zn_2_Cu_4_-α MT1A and Zn_3_Cu_3_-α MT1A), the two species are separated by a slash and the major species is bolded. The presence of key species has been shown in insets.

**Fig. 8:**
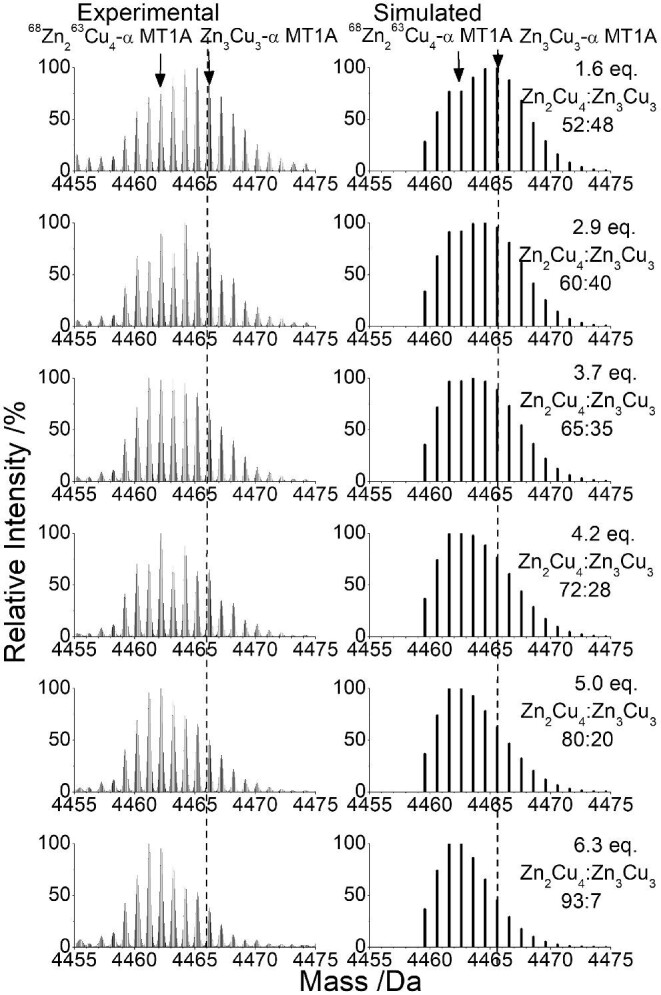
Experimental and simulated deconvoluted ESI-mass spectra showing transition from Zn_3_Cu_3_-α MT1A to Zn_2_Cu_4_-α MT1A as during the ^63^Cu(I) titration of 35 μM ^68^Zn_4_-α MT1A. Full titration is shown in Fig. 7. The dashed line indicates the middle of the initial peak.

*M_4_ to M_5_/M_6_*. Cu(I) addition to Zn_4_-α MT1A leads to the formation of M_5_ and M_6_ species. The M_5_ species that form are Zn_4_Cu_1_-α MT1A and Zn_3_Cu_2_-α MT1A. As more Cu(I) is added, the M_6_ species, Zn_3_Cu_3_-α MT1A (Fig. 7B, shown by the blue arrow, blue ‘6’) is formed. Additional Cu(I) converts the M_6_ Zn_3_Cu_3_-α MT1A to the M_6_ Zn_2_Cu_4_-α MT1A (Fig. 7D, shown by orange arrow, orange ‘6’). Insets have been added to Fig. 7C and D to demonstrate this subtle shift to lower mass; however, there is always a mixture of species as evident by the broad peak. The detailed transition from Zn_3_Cu_3_-α MT1A to Zn_2_Cu_4_-α MT1A is shown in Fig. 8. The experimental MS peak for the mixture of Zn_3_Cu_3_-α MT1A and Zn_2_Cu_4_-α MT1A has been compared with simulations to determine the ratio of the species present at each Cu(I) level.

**Fig. 9:**
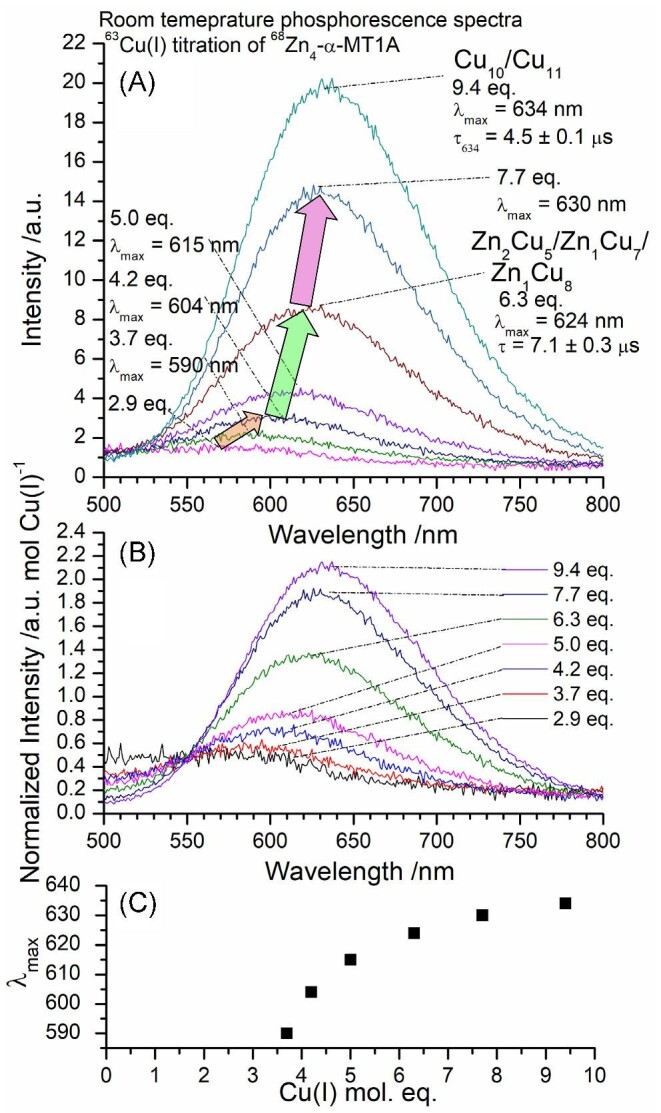
Phosphorescence data for the Cu(I) titration to Zn_4_-α MT1A at pH 7.4 and room temperature. λ_ex_ = 280 nm. (A) Phosphorescence emission spectra showing relative emission intensity. Phosphorescence decay traces are shown in Supplementary Fig. S11. (B) Phosphorescence spectra normalized for the mol. eq. of Cu(I) bound to the protein as determined based on the ESI-mass spectral data are shown in Fig. 7. (C) Trend in λ_max_ as a function of Cu(I) bound to the protein. The mol. eq. refer to the mol. eq. of Cu(I) bound to the protein as determined by ESI-MS.

There is very weak emission with λ_max_ = 590 nm when 2.9 mol. eq. Cu(I) are bound (Fig. 9). We attribute this emission to the formation of Zn_2_Cu_4_-α MT1A (orange arrow).

*M_5_/M_6_ to M_7_/M_8_*. Further addition of Cu(I) increases the total number of metals bound to α MT1A resulting in the M_7_ species, Zn_2_Cu_5_-α MT1A (Fig. 7D, shown by the green arrow, green ‘7’). The metallation plateaus with this species with additional Cu(I), meaning that the abundance of Zn_2_Cu_5_-α MT1A increases and very little of the M_8_ species, Zn_1_Cu_7_-α MT1A, forms until about 6.3 mol. eq. Cu(I) are bound to the protein (Fig. 7G). The formation of Zn_2_Cu_5_-α MT1A increases the emission intensity and results in a redshift in the emission spectra (Fig. 9, green arrow). As the amount of Cu(I) bound to the protein increases, the abundance of the Zn_1_Cu_7_-α MT1A and Zn_1_Cu_8_-α MT1A (pink arrow, pink ‘9’) also increases before the Zn(II) is removed forming Cu_10_-α MT1A and Cu_11_-α MT1A. The results in Figs 4 and 7 clearly show that the two domains have very different binding patterns.

### Significant continuation of phosphorescent intensity at Cu(I): MT ratios >9

The phosphorescence intensity of the orange Cu(I)-thiolate emission measured at room temperature in solution has been characterized as arising from shielded Cu(I)-thiolate clusters. The phosphoresce intensity resulting from Cu(I) binding to the apo protein decreases steeply past Cu(I): MT ratios >7. The expectation is that with >7 Cu(I) bound to the initially apo protein, the cluster structure collapses which exposes the Cu(I) to water, therefore deactivating the triplet state.^60^ As noted in the heading and shown in Fig. 9, when starting from Zn_4_-α MT1A, the phosphorescence intensity is not quenched and actually increases with Cu(I): MT ratios >9.

The changes in metallation are accompanied by shifts in the λ_max_ to higher wavelengths. The phosphorescent lifetimes (Supplementary Fig. S11) are slightly longer than those measured for the β domain fragment, suggesting that the Cu(I) in the α domain is slightly more shielded by the protein backbone. Since there is a mixture of species at each step, the lifetimes obtained are averages for all the species present. For example at the 6.3 eq. step, the Zn_2_Cu_5_-α MT1A, Zn_1_Cu_7_-α MT1A, and Zn_1_Cu_8_-α MT1A species have an average lifetime of 7.1 ± 0.3 µs. Unusually, the emission continues to grow (λ_max_ = 634 nm) even after all of the Zn(II) has been displaced from the protein. This is especially evident when the emission intensity is normalized for the amount of Cu(I) bound (calculated from the ESI-MS) (Fig. 9B). We would expect the cluster structure in the protein to collapse in response to such high levels of Cu(I) and would therefore expect a decrease in the phosphorescence intensity. This effect is seen when Cu(I) is added to the apo α domain fragment.^60^ However, this is not observed when Cu(I) is added to Zn_4_-α MT1A which suggests that the initial presence of the Zn(II) locks the protein backbone in a structure that can accommodate the 10^th^ and 11^th^ Cu(I) in a clustered environment. Even at the end of the titration, the mixture of species has an average phosphorescent lifetime of 4.5 ± 0.1 µs (Supplementary Fig. S11). Based on the X-ray structure of Zn_2_Cd_5_-MT described by Robbins and Stout^82^ where the clustered metals are clearly enveloped by the protein, we conclude that the Cu(I) in the Cu(I)-thiolate clusters is similarly enclosed by the remainder of the protein, thus excluding the solvent from accessing the Cu(I)-S excited state.

Figure 9C shows the trend in the λ_max_ as a function of Cu(I) bound to the protein. There is a gradual redshift in the λ_max_ to higher wavelengths. Notably, there is a lack of intermediary plateaus in the trend unlike the trend for the β domain fragment in Fig. 5C. This may be due to the fact that in the α domain fragment, we always see a mixture of species that gradually shifts upon the addition of Cu(I).

If the binding of Cu(I) to Zn_4_-α MT1A was strictly non-cooperative, in which case Cu(I) binds on a statistical basis to the first available cysteines, one would expect an approximately binomial distribution of species where the centre of the distribution changes to higher metallated species as more metals are added. For example, in a Zn(II) titration of apo α MT1A, an approximately binomial distribution of Zn_2_-α MT1, Zn_3_-α MT1A, and Zn_4_-α MT1A is observed in the ESI-MS when 3.9 mol. eq. of Zn(II) are bound to the protein.^70^ On the other hand, cooperative binding results in the formation of specific species without significant formation of intermediate species. The difference in non-cooperative and cooperative binding is seen in Supplementary Fig. S2 of Melenbacher *et al*.^60^ While it is true that a distribution of species is present at each step in the titration with no single species dominating, there are certain steps in the titration where the speciation deviates from the expected binomial distribution, mainly at the 4.2 mol. eq. point. In this spectrum, after the formation of the M_7_ species, Zn_2_Cu_5_-α MT1A, there is a significant absence of the M_8_ species indicating that the formation of these species is preferred over forming species with higher total number of metals. In contrast to the apo α domain, the high degree of cooperativity is not seen when Zn(II) is present.^60^

### Cu(I) displacement of Zn(II) in Zn_7_-βα MT1A forms mixed metal Zn, Cu species: Zn(II) is not displaced strictly on a stoichiometric or charge basis

**Fig. 10:**
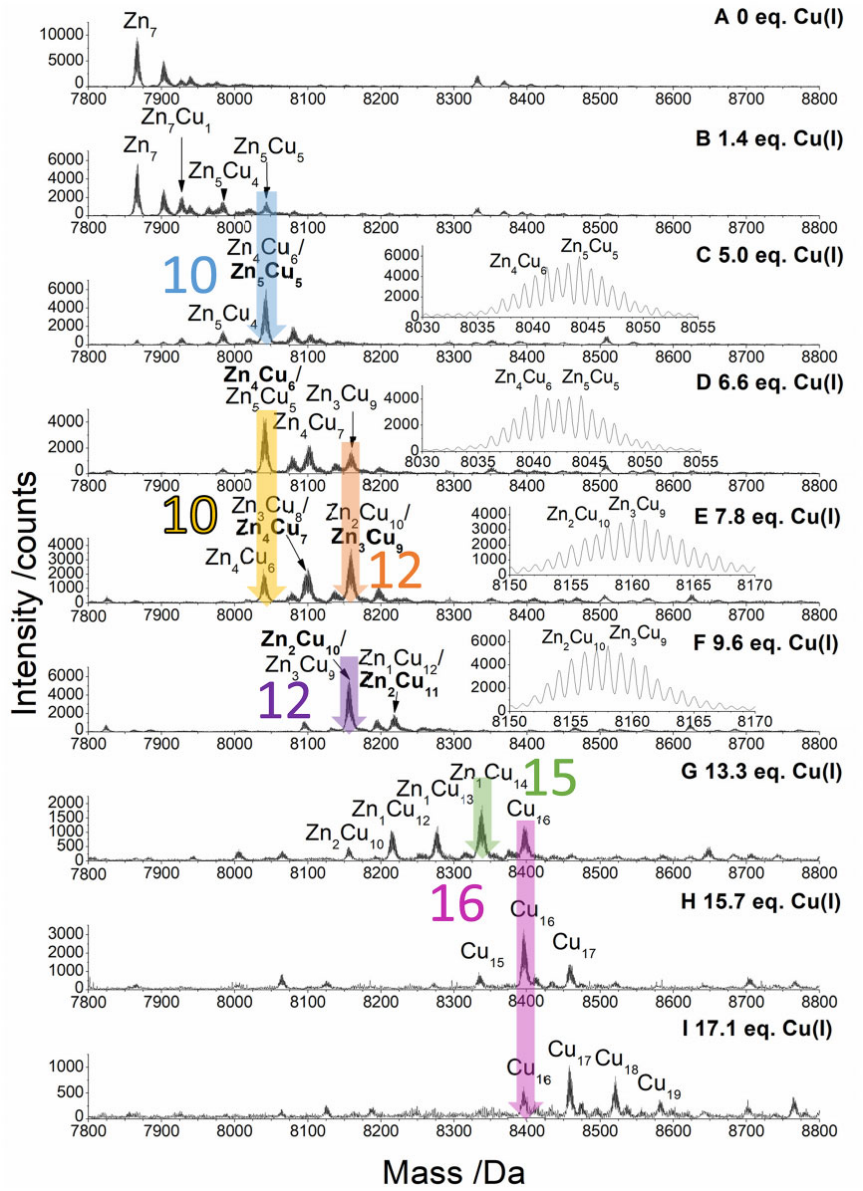
ESI-mass spectra of key steps in the stepwise addition of ^63^Cu(I) into 13.6 μM ^68^Zn_7_-βα MT1A at pH 7.4 and room temperature. The coloured arrows on the mass spectra indicate the key species. Large numbers are shown by the key species to indicate the total number of metals bound. The asterisks denote impurity peaks. All steps in the titration are shown in Supplementary Fig. S12. Charge state data are shown in Supplementary Fig. S13. 3D speciation data are shown in Supplementary Fig. S14. The masses of all species can be found in Supplementary Table S3. The mol. eq. refer to the mol. eq. of Cu(I) bound to the protein as determined by ESI-MS.

**Fig. 11:**
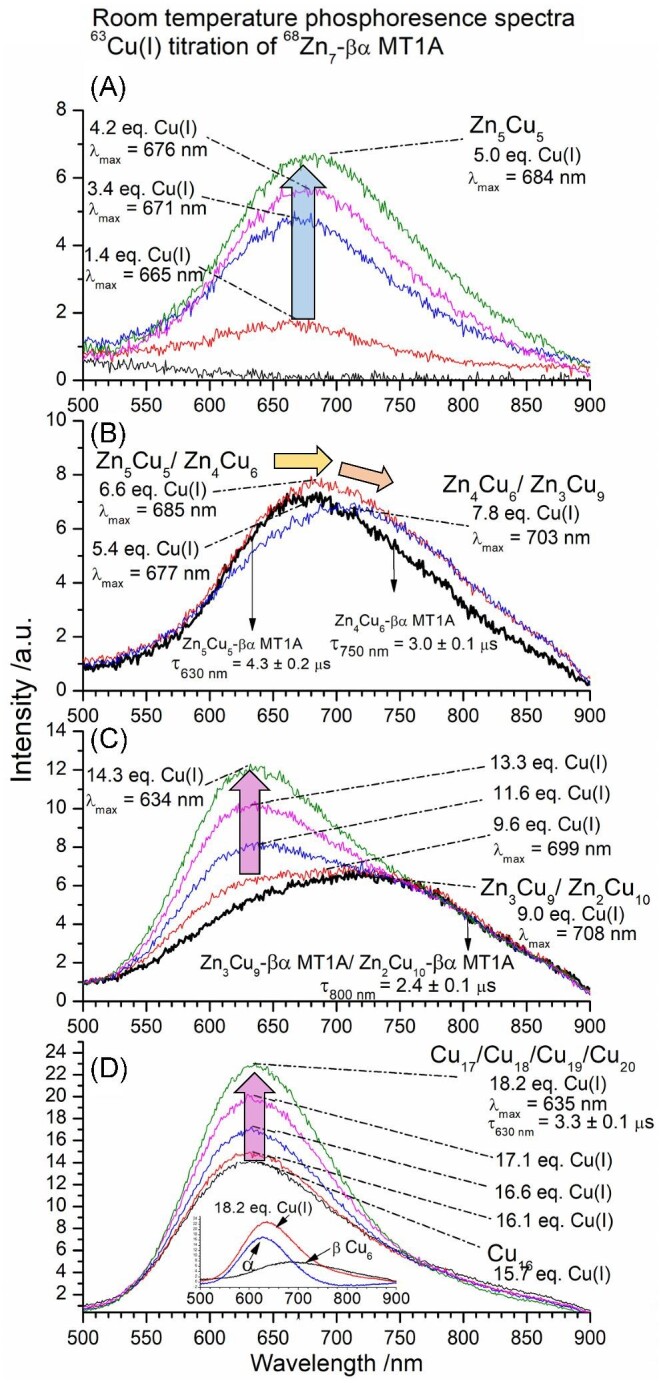
Room temperature phosphorescence spectra measured during the ^63^Cu(I) titration of ^68^Zn_7_-βα MT1A. Cu(I) equivalences have been determined from what is bound according to the ESI-mass spectra data in Fig. 10. (A) Phosphorescence spectra for 1.4–5.0 mol. eq. Cu(I). (B) Phosphorescence spectra for 5.4–7.8 mol. eq. Cu(I). (C) Phosphorescence spectra for 9.0–14.3 mol. eq. Cu(I). (D) Phosphorescence spectra for 15.7–18.2 mol. eq. Cu(I). Phosphorescent decay is shown in Supplementary Fig. S15. The inset in D shows the result of subtracting the 6.6 mol. eq. spectrum (black), characteristic of Cu_6_-β, from the 18.2 mol. eq. spectrum (red) to separate the β domain Cu_6_ emission component (black) from the α domain emission (blue) at 18.2 mol. eq. Cu(I). The specific domain distribution of the Zn(II) and Cu(I) is described in further detail later in the text. The mol. eq. refer to the mol. eq. of Cu(I) bound to the protein as determined by ESI-MS.

^63^Cu(I) was titrated stepwise into a solution of ^68^Zn_7_-βα MT1A at pH 7.4 and room temperature, and the resulting sequence of species was measured by both ESI-MS (Fig. 10) and emission spectroscopy (Fig. 11). In the case where two species result in two adjacent, but resolved peaks in the mass spectrum (e.g., Zn_3_Cu_9_-βα MT1A and Zn_2_Cu_10_-βα MT1A), the two species are separated by a slash and the major species is bolded. Unlike what was assumed in previous work for Zn, Cu exchange, we do not see exchange strictly on a stoichiometric basis (i.e., 1 Cu(I) for 1 Zn(II)) or on a charge basis (2 Cu(I) for 1 Zn(II)). What we mean by this, is that the Zn(II) is retained in the protein longer than expected.

**Fig. 12:**
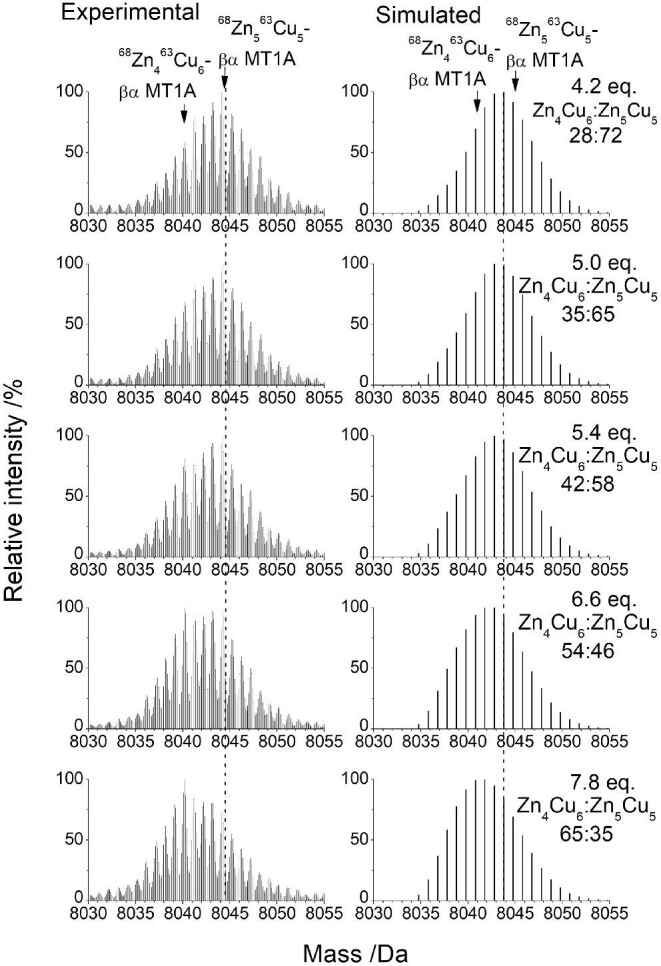
Experimental and simulated deconvoluted ESI-mass spectra showing transition from Zn_5_Cu_5_-βα MT1A to Zn_4_Cu_6_-βα MT1A during the ^63^Cu(I) into 13.6 μM ^68^Zn_7_-βα MT1A. The dashed line indicates the middle of the initial peak.

*M_7_ to M_10_*. Cu(I) binds to the initial M_7_ species, Zn_7_-βα MT1A, which results in a series of species starting with Zn_7_Cu_1_-βα MT1A, Zn_5_Cu_4_-βα MT1A, and Zn_5_Cu_5_-βα MT1A with 1.4 mol. eq. Cu(I) bound (Fig. 10B). There is very weak emission indicating some of the Cu(I) are bound in a shielded environment (Fig. 11A). Further additions of Cu(I) increase the abundances of these species, with Zn_5_Cu_5_-βα MT1A (identified by a dark blue arrow and a blue ‘10’ for the total number of metals bound in Fig. 10C) serving as the first plateau in the titration, meaning that as more Cu(I) is added, the previous species are consumed to form more Zn_5_Cu_5_-MT1A before metallating the protein further. The initial species, Zn_7_-βα MT1A, is still present during the formation of Zn_5_Cu_5_-βα MT1A indicating that the formation of Zn_5_Cu_5_-βα MT1A is preferred over metallating all of the protein to a species with less Cu(I). With the increase in Zn_5_Cu_5_-βα MT1A, the emission intensity increases and the λ_max_ redshifts to 684 nm (Fig. 11A). There is a gradual transition from Zn_5_Cu_5_-βα MT1A to Zn_4_Cu_6_-βα MT1A (Fig. 10E, shown by the yellow arrow and yellow ‘10’ for the total number of metals bound) with the majority of the M_10_ species being Zn_4_Cu_6_-βα MT1A by 6.6 mol. eq. Cu(I) bound. Insets in Fig. 10C and D show an expanded view of the M_10_ peak, though the changes are more evident when examining a series of mass spectra and comparing these changes to simulations (Fig. 12). This allows the exact ratios of Zn_5_Cu_5_-βα MT1A: Zn_4_Cu_6_-βα MT1A to be determined.

The transition from Zn_5_Cu_5_-βα MT1A to Zn_4_Cu_6_-βα MT1A is accompanied by the appearance of a shoulder in the emission band at about 750 nm (Fig. 11B, yellow arrow). As the fraction of Zn_4_Cu_6_-βα MT1A increases, the emission intensity decreases as the cluster within the Zn_4_Cu_6_-βα MT1A species is less emissive than that of the Zn_5_Cu_5_-βα MT1A. Lifetime measurements show that these two species have different phosphorescent lifetimes of 4.3 ± 0.2 μs (Zn_5_Cu_5_-βα MT1A, λ_em_ = 630 nm) and 3.0 ± 0.1 μs (Zn_4_Cu_6_-βα MT1A, λ_em_ = 750 nm) (decay traces shown in Supplementary Fig. S15). The differences in phosphorescent lifetime indicate different degrees of solvent exposure to the cluster. With its longer lifetime, we expect the MT protein to be more tightly wrapped around the Cu(I) containing cluster in Zn_5_Cu_5_-βα MT1A, compared to the Cu(I) cluster in Zn_4_Cu_6_-βα MT1A.

*M_10_ to M_12_*. At 6.6 mol. eq. Cu(I), minor fractions of Zn_4_Cu_7_-βα MT1A and Zn_3_Cu_9_-βα MT1A (Fig. 10D, orange arrow, orange ‘12’ for the total number of metals bound) also form. The M_12_ species Zn_3_Cu_9_-βα MT1A/Zn_2_Cu_10_-βα MT1A are the next plateaus in the titration as we see no further metallated species (M > 12) over the next ∼2 mol. eq. Cu(I) bound (Fig. 10E and F). The distinct lack of a binomial distribution of species, which would occur if binding were strictly on a statistical basis, indicates that the formation of Zn_3_Cu_9_-βα MT1A is slightly preferred over the earlier species as well as further metallation. The formation of Zn_3_Cu_9_-βα MT1A results in a slight decrease in emission intensity and redshift to λ_max_ = 703 nm (Fig. 11B) (orange arrow) due to the loss of the more intense emission band from the Zn_5_Cu_5_-βα MT1A. Overall, the emission spectrum of Zn_3_Cu_9_-βα MT1A is similar to the Zn_4_Cu_6_-βα MT1A emission in the 700–900 nm range.

At 9.6 mol. eq. Cu(I) bound, Zn_2_Cu_10_-βα MT1A (Fig. 10F, purple arrow, purple ‘12’) is now the major species in the solution. The insets in Fig. 10E and F show the change in the ESI-MS. The transition from Zn_3_Cu_9_-βα MT1A to Zn_2_Cu_10_-βα MT1A does not significantly change the emission spectrum in the 700–900 nm range (Fig. 11C). There are slight changes in the emission spectra between 600 and 700 nm suggesting that the transition from the Zn_3_Cu_9_-βα to Zn_2_Cu_10_-βα MT1A may result in a very weakly emitting cluster in the α domain. The lifetime of the Cu(I) cluster in the Zn_3_Cu_9_-βα and Zn_2_Cu_10_-βα MT1A species is 2.4 ± 0.1 μs (λ_em_ = 800 nm) (decay trace shown in Supplementary Fig. S15).

*M_13_ to M_20_*. Also at 9.6 mol. eq., the M_13_ species form, Zn_2_Cu_11_-βα MT1A and Zn_1_Cu_12_-βα MT1A. Remarkably, the final Zn(II) remains bound to the protein with up to 14 Cu(I) ions (Fig. 10G, green arrow). The first Cu(I)-only species to form is Cu_16_-βα MT1A (pink arrow, pink ‘16’) (Fig. 10H). The end of the titration is characterized by approximately binomial distributions of species with up to 20 Cu(I) ions (Fig. 10I). This suggests a non-cooperative binding mode, akin to cysteine modification, which is clearly different from the earlier steps of the titration.

### The Cu(I)-thiolate cluster phosphorescence intensity remains high even with Cu(I): MT ratios >12

It is striking that the emission intensity at 634 nm remains high (Fig. 11C and D), even as more Cu(I) is added. The emission from 750–900 nm is constant after 6.6 mol. eq., which we assigned to a Cu_6_ cluster in the β domain. Therefore, changes in the spectra after 6.6 mol. eq. from 550 to 750 nm are due to metallation of the α domain. The emission spectra in Fig. 11C and D show the formation of a single band with λ_max_ = 634 nm which we suggest to be from the species forming after Zn_2_Cu_10_-βα MT1A up to Cu_20_-βα MT1A (pink arrow). The last species to form, Cu_17_, Cu_18_, Cu_19_, Cu_20_, have a phosphorescent lifetime of 3.3 ± 0.1 μs (λ_em_ = 630 nm, Supplementary Fig. S15).

The inset in Fig. 11D shows the 18.2 mol. eq. spectrum (red) and the result of subtracting the 6.6 mol. eq. spectrum (plotted in black), i.e., the Cu_6_ β emission, to obtain the emission band of the α domain Cu(I) cluster (blue). The emission spectra from the α domain fragment (Fig. 9) are similar to this spectrum in the inset which supports our hypothesis that the α domain is responsible for the continued presence of emission at the end of the titration. This is unlike the Cu(I) titration into apo MT1A^39^ which suggests that the initial presence of the Zn(II) in the α domain aligns the protein in such a way that the increasing number of Cu(I) ions bound occupy a clustered binding site.

### Two different metallation profiles for the β and α domains

**Fig. 13:**
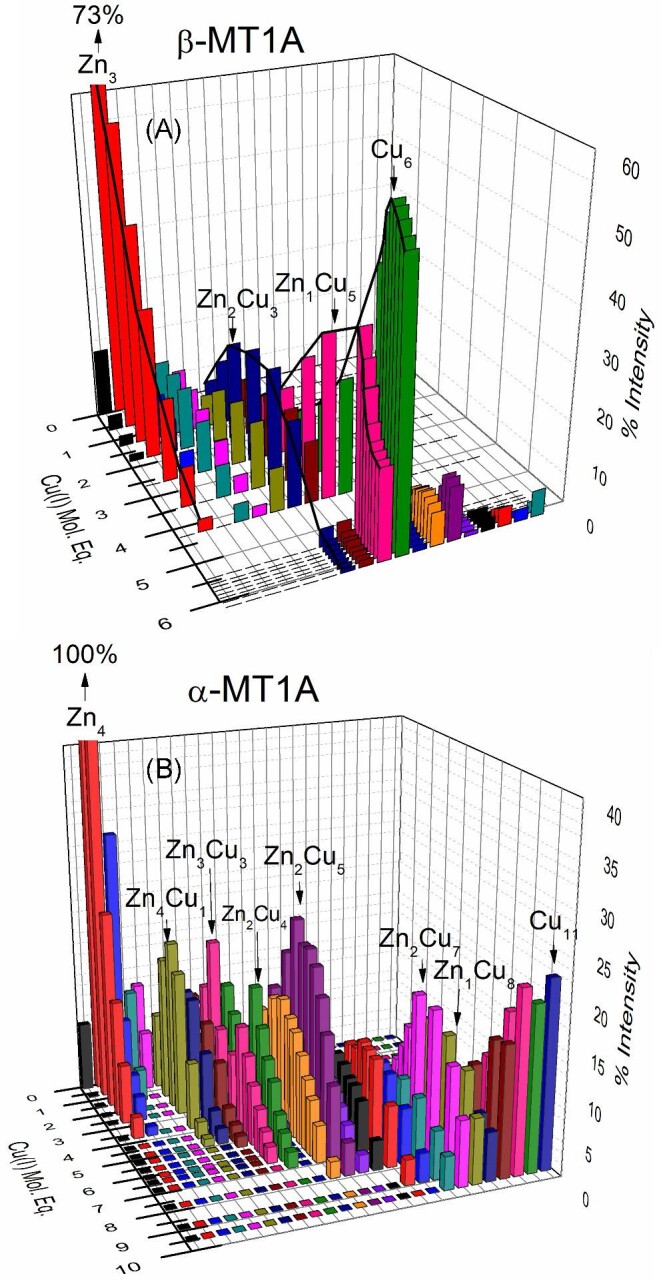
3D speciation showing species forming after the addition of Cu(I) to Zn_3_-β MT1A (A) and Zn_4_-α MT1A (B). Versions of these figures with a full legend are shown in Supplementary Figs S5 and S10. These figures were generated from the ESI-mass spectral data shown in Figs 4 and 7. The mol. eq. refer to the mol. eq. of Cu(I) bound to the protein as determined by ESI-MS. (A) Percent intensity of β domain species, from left to right: Zn_2_-β MT1A, Zn_3_-β MT1A, Zn_4_-β MT1A, Zn_3_Cu_1_-β MT1A, Zn_2_Cu_2_-β MT1A, Zn_3_Cu_2_-β MT1A, Zn_2_Cu_3_-β MT1A, Zn_1_Cu_4_-β MT1A, Zn_1_Cu_5_-β MT1A, Cu_6_-β MT1A, Zn_2_Cu_5_-β MT1A, Zn_1_Cu_6_-β MT1A, Cu_7_-β MT1A, Zn_2_Cu_8_-β MT1A, Zn_1_Cu_7_-β MT1A, Cu_8_-β MT1A, Zn_1_Cu_8_-β MT1A, Cu_9_-β MT1A. (B) Percent intensity of α domain species, from left to right: Zn_3_-α MT1A, Zn_4_-α MT1A, Zn_3_Cu_1_-α MT1A, Zn_2_Cu_2_-α MT1A, Zn_5_-α MT1A, Zn_4_Cu_1_-α MT1A, Zn_3_Cu_2_-α MT1A, Zn_3_Cu_3_-α MT1A, Zn_2_Cu_4_-α MT1A, Zn_1_Cu_5_-α MT1A, Zn_2_Cu_5_-α MT1A, Zn_1_Cu_6_-α MT1A, Zn_3_Cu_5_-α MT1A, Zn_2_Cu_6_-α MT1A, Zn_1_Cu_7_-α MT1A, Zn_3_Cu_6_-α MT1A, Zn_2_Cu_7_-α MT1A, Zn_1_Cu_8_-α MT1A, Zn_2_Cu_8_-α MT1A, Zn_1_Cu_9_-α MT1A, Cu_10_-α MT1A, Zn_1_Cu_10_-α MT1A, Cu_11_-α MT1A.

Figure 13 shows the % abundance of each species as a function of Cu(I) bound to the protein where the relative intensities were determined from the ESI-MS data (Figs 4 and 7). The use of this 3D block diagram format separates the sequential formation of species in both the X and Z directions such that the stoichiometric changes taking place as a function of Cu(I) bound to the protein can be clearly observed. This figure emphasizes the fact that there are two different metallation pathways for the β MT1A (Fig. 13A) and α MT1A (Fig. 13B) domain fragments. We see only a few species forming in the β domain fragment, specifically Zn_2_Cu_3_-β MT1A, then Zn_1_Cu_5_-β MT1A, and finally Cu_6_-β MT1A as the major final product.

The α domain fragment is characterized by a distribution of mixed Zn, Cu species that are all present throughout the titration at relatively similar abundances. Particularly, Zn(II) is present in the α domain fragment up to Zn_1_Cu_8_-α MT1A. Further Cu(I) results in displacement of the final Zn(II) and formation of up to Cu_11_-α MT1A.

Overall, these diagrams show how the β domain has maximum saturation with 6 Cu(I), whereas the α domain binds to form a number of species with a maximum of 11 Cu(I). We speculate that the formation of the Cu_6_ cluster in the β domain may be strengthened by the occurrence of the four Cys-X-Cys motifs (Fig. 1) in the β domain which may provide a more systemic series of donor ligands. The additional cysteines in the α domain appear to stabilize the presence of the Zn(II) together with the Cu(I). In the absence of the Zn(II), the maximum cluster size in the α domain fragment was found to be Cu_7_S_9_.^60^ The key features are that in the βα MT1A (to be described below), the species forming in the β domain, for example Cu_6_-β, and the species in the α domain, for example Zn_1_Cu_8_-α, exist together (Table 3). In particular, the mixture of the Zn, Cu ratios in the α domain can only be determined using the isotopically pure ^68^Zn(II) and ^63^Cu(I) because of the extensive overlap of the natural abundance isotopes.

**Table 3.** Summary of the major species formed following ^63^Cu(I) metallation of Zn_3_-β MT1A, Zn_4_-α MT1A, and Zn_7_-βα MT1A and their emission band centres

β domain fragment species	Cationic charge	β emission band centre	α domain fragment species	Cationic charge	α emission band centre	Total stoichiometry as seen in βα MT1A experimental data^a^	Cationic charge	Emission band centre
Zn_3_Cu_1_-β MT1A	7	-	Zn_4_-α MT1A	8	-	Zn_7_Cu_1_-βα MT1A	15	
Zn_1_Cu_5_-β MT1A	7	700 nm	Zn_4_-α MT1A	8	-	Zn_5_Cu_5_-βα MT1A	15	684 nm
Cu_6_-β MT1A	6	738 nm	Zn_4_-α MT1A	8	-	Zn_4_Cu_6_-βα MT1A	14	750 nm
Cu_6_-β MT1A	6	738 nm	Zn_4_Cu_1_-α MT1A	9	-	Zn_4_Cu_7_-βα MT1A	15	750 nm
Cu_6_-β MT1A	6	738 nm	Zn_3_Cu_2_-α MT1A	8	-	Zn_3_Cu_8_-βα MT1A	14	750 nm
Cu_6_-β MT1A	6	738 nm	Zn_3_Cu_3_-α MT1A	9	-	Zn_3_Cu_9_-βα MT1A	15	750 nm
Cu_6_-β MT1A	6	738 nm	Zn_2_Cu_4_-α MT1A	8	590 nm	Zn_2_Cu_10_-βα MT1A	14	750 nm
Cu_6_-β MT1A	6	738 nm	Zn_2_Cu_5_-α MT1A	9	624 nm	Zn_2_Cu_11_-βα MT1A	15	634 nm
Cu_6_-β MT1A	6	738 nm	Zn_1_Cu_8_-α MT1A	10	634 nm	Zn_1_Cu_14_-βα MT1A	16	634 nm

^a^The species shown in this column are observed in the ESI-mass spectral data shown in Fig. 10 and are the sum of the isolated domain fragment species.

### Unexpected retention of the Zn(II)

The presence of this single Zn(II) with its binding constant^83^ of 10^11^ in the presence of a huge stoichiometric excess of Cu(I) with its binding constant of 10^19^ is totally unexpected. The 10^19^ value for MT is based on the K_F_ value for the formation of Cu_10_-MT2 determined by Banci *et al*.^9^ and updated with a more accurate DTT binding constant from Xiao *et al*.^84^ In previous studies, analysis of the spectral data assumed that Cu(I) would systematically displace the Zn(II) on a stoichiometric or charge basis.^68,71,72^ Even in studies using ESI-MS methods, the presence of the Zn(II) was disguised by the significant overlap of the natural abundance isotopic masses.^73–75^

The presence of Zn(II) in βα MT1A increases the number of stable species that form at physiological pH. We have previously reported that Cu(I)-thiolate species containing specifically 4, 6, 10, and 13 Cu(I) ions are formed from apo-βα MT1A with few intermediate species.^39,60^ In the cell, it is likely that Cu(I) will be binding to partially metallated Zn(II)-MT or newly synthesized apo-MT. The presence of multiple stable Zn, Cu species cements the idea that MT is a good candidate for Zn(II) and Cu(I) storage as this would require flexibility in the stoichiometry. Most species form at a relatively similar abundance, suggesting that the energy minimum for this group of species is very broad.

### Use of the β domain and α domain fragment data to analyse βα speciation: metallation of the Zn_3_-β and Zn_4_-α fragments with Cu(I) match the metallation pathway of the Zn_7_-βα MT1A

We can gain insight into the specific clusters that form in each domain of the Zn, Cu-βα MT1A species by analysing the ESI-MS metallation and emission profiles of the Zn_3_-β-MT1A and Zn_4_-α MT1A domain fragments (Table 3). We have previously used this method with the apo protein.^60^ The use of the fragments gives valuable insight into the domain structures of the Zn, Cu-βα MT1A species in the absence of NMR or X-ray structures. The key species are summarized graphically in Supplementary Fig. S16.

*Cu(I) binds first to the β domain leaving Zn_4_-α*. Previously, we have shown that Cu(I) preferentially binds to the apo β domain over the apo α domain.^60^ Assuming Cu(I) also preferentially binds to the β domain of Zn_7_-βα MT1A, the species that form in the Zn_3_-β domain fragment upon addition of Cu(I) combined with Zn_4_ in the α domain accounts for the species that form in the first half of the titration of Zn_7_-βα MT1A. For example, Zn_3_Cu_1_, Zn_1_Cu_5_, and Cu_6_ in the β domain and an intact Zn_4_ cluster in the α domain would result in the stoichiometries seen in the full protein: Zn_7_Cu_1_-βα MT1A, Zn_5_Cu_5_-βα MT1A, and Zn_4_Cu_6_-βα MT1A. This is supported by the similarities in the Zn_5_Cu_5_-βα MT1A (684 nm) and Zn_1_Cu_5_-β MT1A (700 nm) as well as similarities in the Zn_4_Cu_6_-βα MT1A (750 nm) and Cu_6_-β MT1A (738 nm) emission spectra. We therefore attribute the emission in the Zn_5_Cu_5_-βα MT1A (684 nm) and Zn_4_Cu_6_-βα MT1A (750 nm) species to be from a Zn_1_Cu_5_ and Cu_6_ cluster, respectively, in the β domain with a non-emissive Zn_4_ cluster in the α domain. The trend in the relative emissiveness of the transition from Zn_5_Cu_5_-βα MT1A to Zn_4_Cu_6_-βα MT1A also tracks the trend in emissiveness seen in the β domain fragment. The Cu_6_ cluster formed from the apo β domain fragment also has a similar emission spectrum.^60^

*After β domain is saturated with 6 Cu(I), the α domain binds Cu(I)*. The results from the Cu(I) metallation of Zn_3_β-MT1A (Fig. 4) suggest that after forming the Cu_6_ cluster within the β domain of βα MT1A, the β domain cannot accommodate more Cu(I). Therefore, one would expect that the formation of species after Zn_4_Cu_6_-βα MT1A is due to the metallation of the α domain. The species forming in α-MT1A, Zn_4_Cu_1_, Zn_3_Cu_2_, and Zn_3_Cu_3_, and Zn2Cu4, combined with Cu_6_ in the β domain explain the stoichiometry of the remaining βα species, Zn_4_Cu_7_-βα MT1A, Zn_3_Cu_8_-βα MT1A, Zn_3_Cu_9_-βα MT1A, and Zn_2_Cu_10_-βα MT1A. The mass spectra in Figs 7C and 10E as well as Fig. 7H and G have similar profiles providing further evidence that metallation of the α domain is responsible for this part of the titration. The emission spectra of the Zn_3_Cu_9_-βα MT1A and Zn_2_Cu_10_-βα MT1A resemble the spectrum for Zn_4_Cu_6_-βα MT1A suggesting that the emitting cluster for all of these species is a Cu_6_ cluster in the β domain. The Zn_3_Cu_9_-βα MT1A/Zn_2_Cu_10_-βα MT1A mixture also has the same phosphorescent lifetime as the Cu_6_ cluster formed in the β domain fragment. While the phosphorescent lifetime of the Cu_6_ cluster in the Zn_4_Cu_6_-βα MT1A (measured at 5.4 mol. eq. Cu(I)) appears to decrease for the same Cu_6_ cluster in the Zn_3_Cu_9_-βα MT1A and Zn_2_Cu_10_-βα MT1A species, the lifetime measured at 5.4 mol. eq. of Cu(I) may appear longer due to overlap of the emission bands. A small amount of emission from the Zn_5_Cu_5_-βα MT1A (which has a longer lifetime) at the same wavelength as the Zn_4_Cu_6_-βα MT1A may be present, therefore inflating the measured lifetime. For Zn_3_Cu_9_-βα MT1A and Zn_2_Cu_10_, with an emissive Cu_6_ cluster in the β domain, this leaves Zn_3_Cu_3_ and Zn_2_Cu_4_ in the α domain which appear to be non-emissive. However, species forming with M > 12 have emission at 634 nm, which is characteristic of the α domain emission. This indicates that emissive clusters form in the α domain with a combination of Cu(I) and Zn(II). For example, the last Zn(II) containing species, Zn_1_Cu_14_, is formed as a result of a Cu_6_ cluster in the β domain and a mixed metal Zn_1_Cu_8_ cluster in the α domain.

### The total cationic charge is surprisingly constant while Cu(I) is added to Zn(II)-MT

Examining the cationic charge from the metals in each Zn, Cu-βα MT1A species reveals that most species have a combination of metals that sums to a charge of 14 + to 16+ (Supplementary Fig. S17) with only one species having a cationic charge of <14+. We suggest that species with this range of cationic charge may be more stable and Zn(II) is retained in the cluster region until enough Cu(I) has been added to maintain the charge within this region of stability. The charges of the species forming in the β and α domain fragments can also be calculated and added together to model the full βα-MT1A protein (Table 3). This analysis assumes that the Cu(I) displaces Zn(II) in the β domain first, leaving the four Zn(II) ions in the α domain with an 8+ charge. Once the β domain forms Cu_6_, the charges for the α domain species are added to the 6+ charge of the Cu_6_ cluster in the β domain. At each combination of β and α domain fragment species, the summed charge is equal to the charge of the species measured experimentally in the full βα-MT1A protein. For example, the 6+ charge of Cu_6_ and the 8+ charge of Zn_4_ sum together to the 14+ charge of Zn_4_Cu_6_-βα MT1A. This provides strong supporting evidence that the stoichiometries of the clusters forming in each domain within the full βα-MT1A protein systematically follow the species that form in the individual domain fragments. The species with charges of 17+ to 20+ are less stable and occur when the binding pattern has shifted to a non-specific thiolate binding pathway, akin to cysteine modification, as evident by the binomial distributions of species.

### Our results in context with previous reports

#### Use of the ESI-MS for metallation studies

ESI-MS provides details of all species in solution allowing for the determination of the exact stoichiometry of the possibly many species that exist at each step in the titration. The data in this paper illustrate the complexity of metallation with two different metals, but there is also complexity in the metallation of MTs with a single metal. In the past, the extent of metallation was often determined using atomic absorption spectrometry (AAS) or inductively coupled plasma atomic emission spectrometry (ICP-AES). More recently ICP-MS has also been used. Without the separation of each metallated species, which is not typically carried out before analysis, these techniques provide an average of the metallation status such that there is no information on the distribution of the metals in different species. This can be misleading when there are multiple species present or the presence of cooperativity. We now discuss some previous results of Zn, Cu-MTs that were found before ESI-MS was widely available. For example, Chen *et al*.^38,65^ quantify Cd, Zn, and Cu in rat kidney MT using AAS. They conclude that the MT1 isolated from rat kidney cytosol contains a 1:1 ratio of Cu: Zn.^65^ Our ESI-MS results suggest that either Zn_5_Cu_5_-βα MT1A or Zn_4_Cu_6_- βα MT1A could be possibilities for the MT stoichiometry in the kidney as these are the only species we find with a 1:1 ratio. However, our results only take account of the species that form when Cu(I) is added to Zn_7_-MT1A, which may not be the case *in vivo*. Similarly, Zn, Cu-MT1/2 has been extracted from calf liver samples. Chen *et al*.^38^ reported a range of Cu: Zn ratios by AAS methods. Notably, a sample containing 9.6 g atoms of metal/mol protein had a Cu: Zn ratio of 1.4, which would be similar to the Zn_4_Cu_6_-βα MT1A species we report.

Our results on Cu(I) binding to the β domain fragment of human MT1A can be compared to the previous studies of the β domain fragment of mouse MT1.^72^ Bofill *et al*. report the cooperative formation of a Zn_1_Cu_4_ cluster in the β domain fragment of mouse MT1.^72^ In this study, the metal content was also determined by AAS. In our results, we see multiple species continuously throughout the titration and only minor amounts of this Zn_1_Cu_4_-β MT1A species. With only the average metal binding available from the AAS data, it is possible that a fraction of the protein measured by Bofill *et al*.^72^ may have had more than 4 Cu(I) bound. This is seen in our studies of the human β MT1A domain fragment where with an average of 4.2 Cu(I) ions bound to the protein, a variety of species are seen with most having 3–5 Cu(I) ions bound.

We can also compare our α domain fragment and βα results with the species reported to form from natural abundance Zn_4_-α mouse MT and Zn_7_- mouse MT by Bofill *et al*.^73^ The three species reported to form from Zn_4_-α mouse MT after the addition of natural abundance Cu(I) are Zn_2_Cu_3_-α mouse MT, Zn_1_Cu_4_-α mouse MT, and Zn_1_Cu_5_-α mouse MT. We do not see species with these stoichiometries forming, rather at the point at which 5 or 6 metals are bound to the human α MT1A, we see mainly Zn_4_Cu_1_-α MT1A, Zn_3_Cu_3_-α MT1A, and Zn_2_Cu_4_-α MT1A (Fig. 7). In addition, Bofill *et al*.^73^ report several Zn, Cu βα species, including Zn_3_Cu_7_-MT. From our results reported in Fig. 10, we find that when 10 metals are bound to the protein, there are still 5 Zn(II) ions present in human βα MT1A. These differences in stoichiometric ratios may be due to the methods used to quantify the stoichiometry. While ESI-MS was used by Bofill *et al*.,^73^ the closeness of the Cu(I) and Zn(II) natural abundance masses required the additional use of ICP-AES to determine stoichiometry: Chelex-100 was used to bind the displaced Zn(II) and then the metals bound to the protein were quantified using ICP-AES.^73^ This technique results in the average metal content in the solution, which again makes determination of specific stoichiometric ratios very difficult.

#### The importance of using isotopically pure elements

With the use of isotopically pure ^63^Cu(I) and ^68^Zn(II), we have resolved the longstanding difficulty of determining the exact stoichiometry of mixed Zn, Cu-MT species. The use of isotopically pure elements has been used successfully in previous MS studies of proteins. For example, in a study by Blindauer *et al*.^77^  ^67^Zn(II) was used to study Zn(II) exchange in the Zn_4_ cluster of cyanobacterial MT. Only one Zn(II) ion was found to be inert to exchange.^77^ Meloni and Vašák^78^ used isotopically pure ^63^Cu(I) and ^67^Zn(II) and reported the removal of Cu(II) from α synuclein-^63^Cu(II) by MT3. More recently, Crack *et al*.^79^ used isotopically pure ^34^S to label the sulphide ions of FeS clusters which allowed the unambiguous assignment of the FeS cluster stoichiometry. Together, these studies illustrate the dramatic increase in information that is obtained using a combination of ESI-MS methods and isotopically pure elements.

## Conclusions

The novel use of isotopically pure ^68^Zn(II) and ^63^Cu(I) eliminates the overlapping isotopic patterns of natural abundance Zn(II) and Cu(I) which is required to determine the stoichiometry of mixed metal Zn, Cu-MT species. This, in combination with MS simulations, reduces the difficulty in analysing the MS data of mixed metal Zn, Cu-MT species, allowing for the exact determination of the stoichiometry of all Zn, Cu-MT species that form upon the addition of ^63^Cu(I) to ^68^Zn_7_-βα MT1A. Room temperature solution phosphorescence measurements, combined with ESI-MS measurements, allow the unambiguous identification of multiple mixed Zn, Cu-thiolate clusters in MT1A that exhibit specific λ_max_ and μs lifetimes.

We used the β and α domain fragment room temperature emission spectra in parallel with the stepwise ESI-MS data to guide the assignment of the βα emission spectra. The addition of Cu(I) to Zn_7_-βα MT1A resulted in the formation of Zn_5_Cu_5_-βα MT1A, which exhibits emission at 684 nm due to a Zn_1_Cu_5_ cluster in the β domain. The appearance of a shoulder at 750 nm with the formation of the Zn_4_Cu_6_-βα MT1A species is due to the formation of a Cu_6_ cluster, with the Cu(I) again located solely in the β domain. Addition of Cu(I) to form Zn_3_Cu_9_-βα MT1A and Zn_2_Cu_10_-βα MT1A resulted in very little change in the emission spectra, indicating the continued presence of the Cu_6_-β cluster. The additional Cu(I) clearly binds to the α domain and is either not emissive or very weakly emissive at this stage. Further metallation resulted in an increase in emission intensity at 634 nm that arises from clusters forming in the α domain. Even at the end of the titration, emission spectra characteristic of Cu(I)-thiolate clusters were observed, which is quite unlike Cu(I) metallation of the apo MT1A protein with molar excess Cu(I).^39^ The emission intensity remains high at the end of the titration with the Zn_4_-α MT1A domain fragment or Zn_7_-βα MT1A, but not for the Zn_3_-β MT1A domain fragment. The initial presence of the Zn(II) must have a profound effect on the overall structure of the α domain. We suggest that the Zn(II) locks the α domain in a specific conformation that allows for the Zn, Cu(I)-thiolate cluster structure and later the pure Cu(I)-thiolate cluster structure to be maintained even in the presence of molar excess Cu(I).

Finally, we combined all of the species forming in the Zn_3_-β MT1A and Zn_4_-α MT1A domain fragments upon Cu(I) addition to identify each domain's contribution to the overall stoichiometry of species forming in the Zn_7_-βα MT1A protein. The species that formed in the Zn_3_-β MT1A domain fragment also form in the full βα MT1A protein while the α domain remains as Zn_4_. This accounts for all the species that form in the first half of the titration of Cu(I) into Zn_7_-βα MT1A. The species that formed in the Zn_4_-α MT1A domain fragment then form in βα MT1A in combination with the Cu_6_ cluster that has already formed in the β domain of the βα-MT1A which accounts for all the species forming in βα MT1A in the second half of the titration. Further, we have examined the cationic charge brought in by the metals in each species and have found that Cu(I) binding to Zn_7_-βα MT1A protein occurs over a narrow, stable region of 14+ to 16+. The cationic charge of the species that form in the β domain combined with the cationic charge of Zn_4_-α combine to the cationic charges of the species forming in the first half of the βα titration. The cationic charge of the Cu_6_ cluster in the β domain combined with the cationic charges of the α domain species add up to the cationic charges of the species formed in the second half of the βα titration.

## Supplementary Material

mfac101_Supplemental_File

## Data Availability

The data underlying this article are available in the article and in its online supplementary material.
