# Supplementary material for: 63Cu(I) binding to human kidney 68Zn7-βα MT1A: determination of Cu(I)-thiolate cluster domain specificity from ESI-MS and room temperature phosphorescence spectroscopy
Source: Metallomics. 2022 Dec 30;15(1):mfac101. doi: 10.1093/mtomcs/mfac101 (PMC9846682; doi:10.1093/mtomcs/mfac101)
Supplement: mfac101_Supplemental_File [file mfac101_supplemental_file.pdf]

^{63}Cu(I) binding to human kidney $^{68}Zn_7$-βα MT1A: Determination of Cu(I)-thiolate cluster domain specificity from ESI-MS and room temperature phosphorescence spectroscopy: Supplementary Data

Adyn Melenbacher[a], Lina Heinlein[a, b], Andrea Hartwig[b], and Martin J. Stillman[*a]

[a]Department of Chemistry, The University of Western Ontario, 1151 Richmond St., London, ON, N6A 5B7, Canada

[b]Department of Food Chemistry and Toxicology, Institute of Applied Biosciences (IAB), Karlsruhe Institute of Technology (KIT), Adenauerring 20a, 76131, Karlsruhe, Germany.

Corresponding Author: Martin J. Stillman

Department of Chemistry, The University of Western Ontario, 1151 Richmond St., London, ON, N6A 5B7, Canada

Characterizing mixed-metal ^{68}Zn,^{63}Cu-MTs

Determining the necessity of both isotopically pure ^{63}Cu(I) and ^{68}Zn(II)

Even with a smaller number of metals ions and the small β domain fragment, Figure S1 demonstrates experimentally that both isotopically pure ^{63}Cu(I) and ^{68}Zn(II) are necessary to resolve the stoichiometry of the Zn,Cu-MT species formed. The use of isotopic ^{68}Zn(II) in combination with natural abundance Cu(I) results in a small shift in the peak centre from 4131 Da to 4128.5 Da (Fig. S1A, Fig, S2A,B). The identities of the species in these two envelopes are $^{68}Zn_1Cu_5$-β MT1A and Cu_6-β MT1A (Fig. S1A). In the absence of isotopic ^{63}Cu, these two envelopes are confirmed to be different species by their emission (Fig. S1B) and circular dichroism spectra (Fig. S1C). Without the ^{68}Zn(II), the peaks for each species would be even broader from the greater degree of overlap due to the presence of multiple Zn(II) and Cu(I) isotopes. This has been an issue that previous researchers studying Zn,Cu-MTs faced when using ESI-mass spectrometric methods.

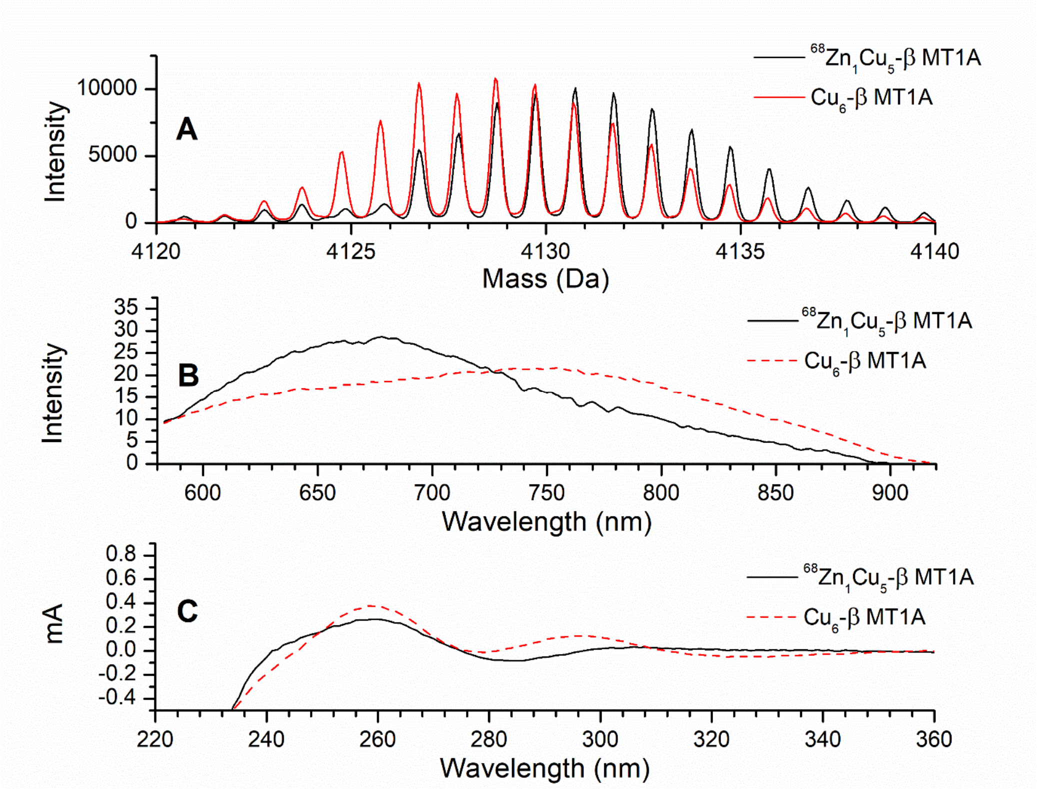

Figure S1 ESI-mass spectral data (A), phosphorescence spectra (B), and circular dichroism spectra (C) for $^{68}Zn_1Cu_5$-β MT1A and Cu_6-β MT1A. Note that the Cu(I) used in this experiment was natural abundance Cu(I).

The data are far more resolved with the use of both ^{68}Zn and ^{63}Cu (as shown by the blue and green rectangles in Fig. S2), which further increases the separation between the two peaks allowing for the complete and unambiguous determination of the Cu(I) and Zn(II) stoichiometry. The envelope for $^{68}Zn_1{}^{63}Cu_5$-β MT1A is centred at 4128.5 Da (Fig. S2C) whereas the $^{63}Cu_6$-β MT1A has a peak maximum at 4124.5 Da (Fig. S2D). It is not surprising that previous attempts to determine the values of n and m in the mixed-metal, Cu_nZn_m-MT species, were unsuccessful when using Zn(II) and Cu(I) of natural abundance.

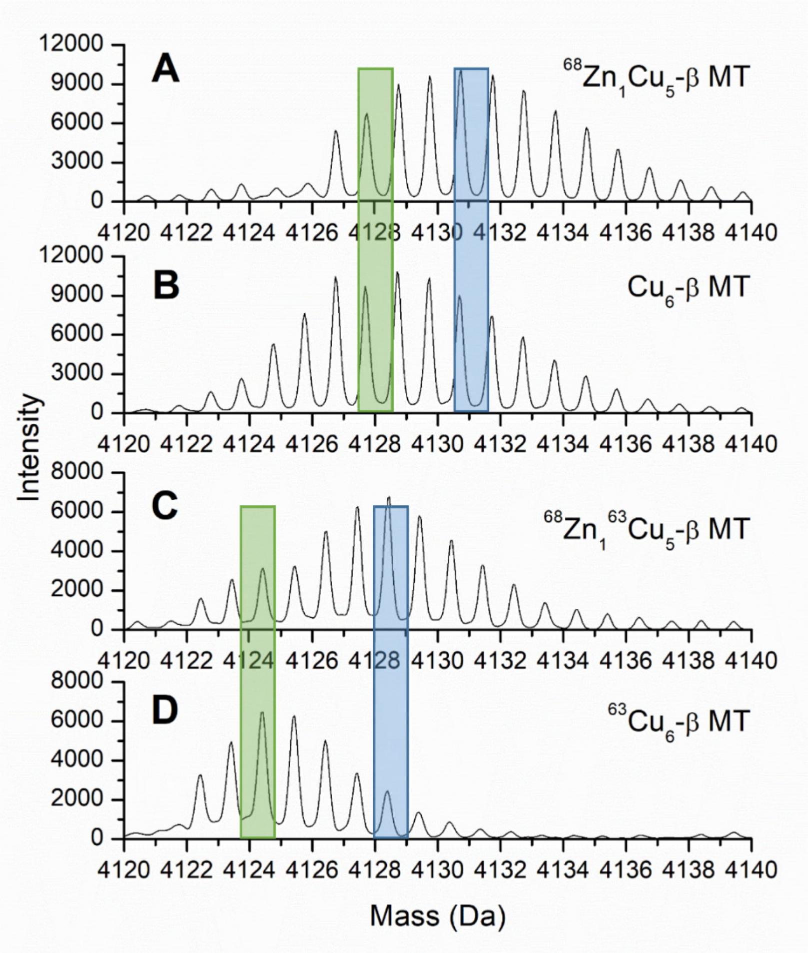

Figure S2 Deconvoluted mass spectral data of ^{68}Zn,Cu-β MT.

Comparison of A. $^{68}Zn_1Cu_5$-β MT (peak maximum at 4131 Da) with B. Cu_6-β MT (peak maximum at 4128.5 Da) using natural abundant Cu(I).

Comparison of C. $^{68}Zn_1{}^{63}Cu_5$-β MT (peak maximum at 4128.5 Da) with D. $^{63}Cu_6$-β MT (peak maximum at 4124.5 Da) using isotopically pure ^{63}Cu(I).

$^{63}Cu(I)$ Addition to $^{68}Zn_3$-β MT1A at pH 7.4 and room temperature

Table S1: Mass of-species forming upon the addition of $^{63}Cu(I)$ to $^{68}Zn_3$-β MT1A

Species	Mass (Da)
apo β MT1A	3752
$^{68}Zn_2$-β MT1A	3884
$^{68}Zn_3$-β MT1A	3950
$^{68}Zn_3{}^{63}Cu_1$- β MT1A	4012
$^{68}Zn_2{}^{63}Cu_2$- β MT1A	4008
$^{68}Zn_2{}^{63}Cu_3$- β MT1A	4070
$^{68}Zn_1{}^{63}Cu_4$- β MT1A	4066
$^{68}Zn_1{}^{63}Cu_5$- β MT1A	4128
$^{63}Cu_6$- β MT1A	4124
$^{68}Zn_1{}^{63}Cu_6$- β MT1A	4190
$^{63}Cu_7$- β MT1A	4186

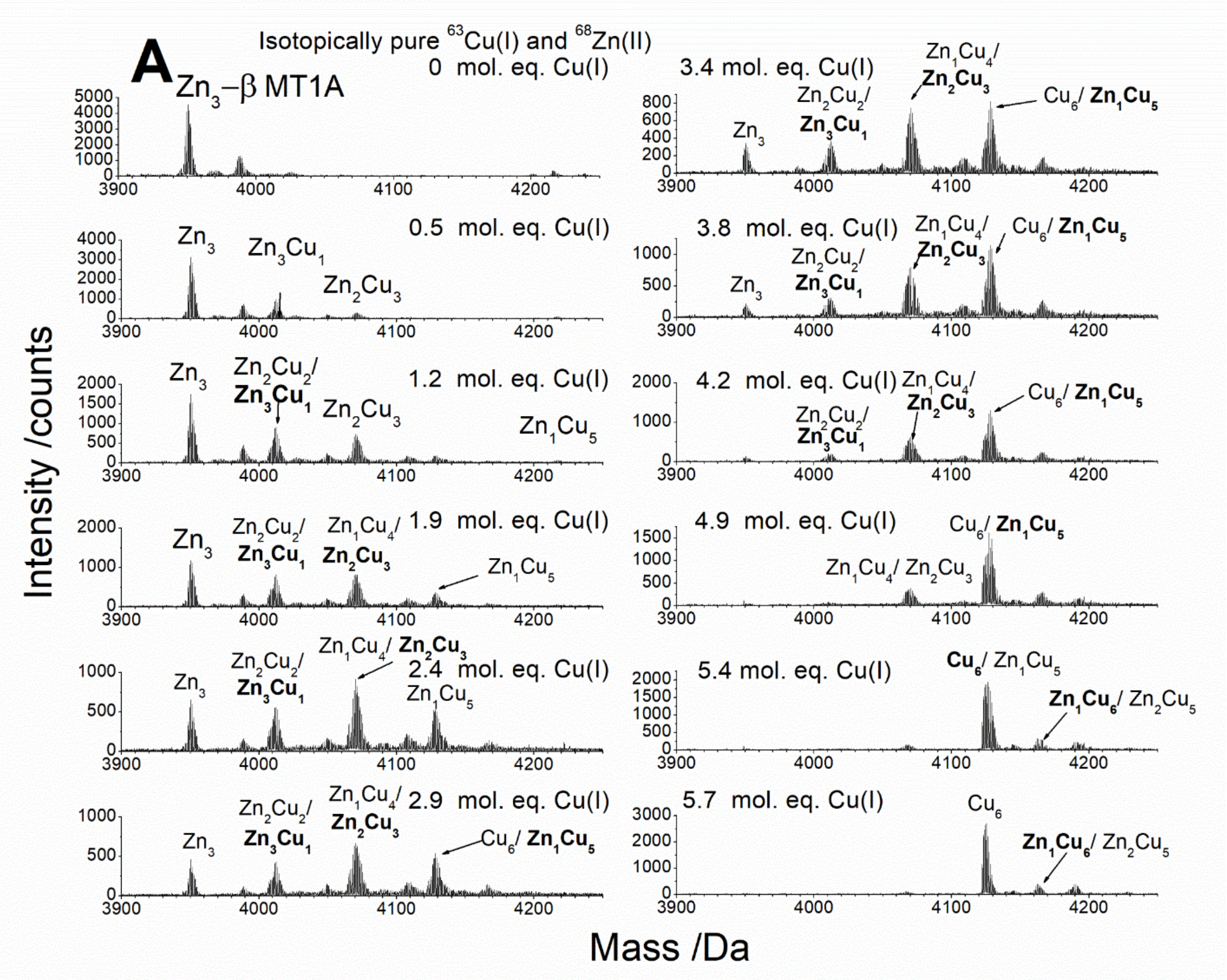

Figure S3 All deconvoluted mass spectra for the titration shown in Fig. 4 of $^{63}Cu(I)$ added to 29.6 µM $^{68}Zn_3$-β MT1A. The titration was carried out at room temperature and pH 7.4.

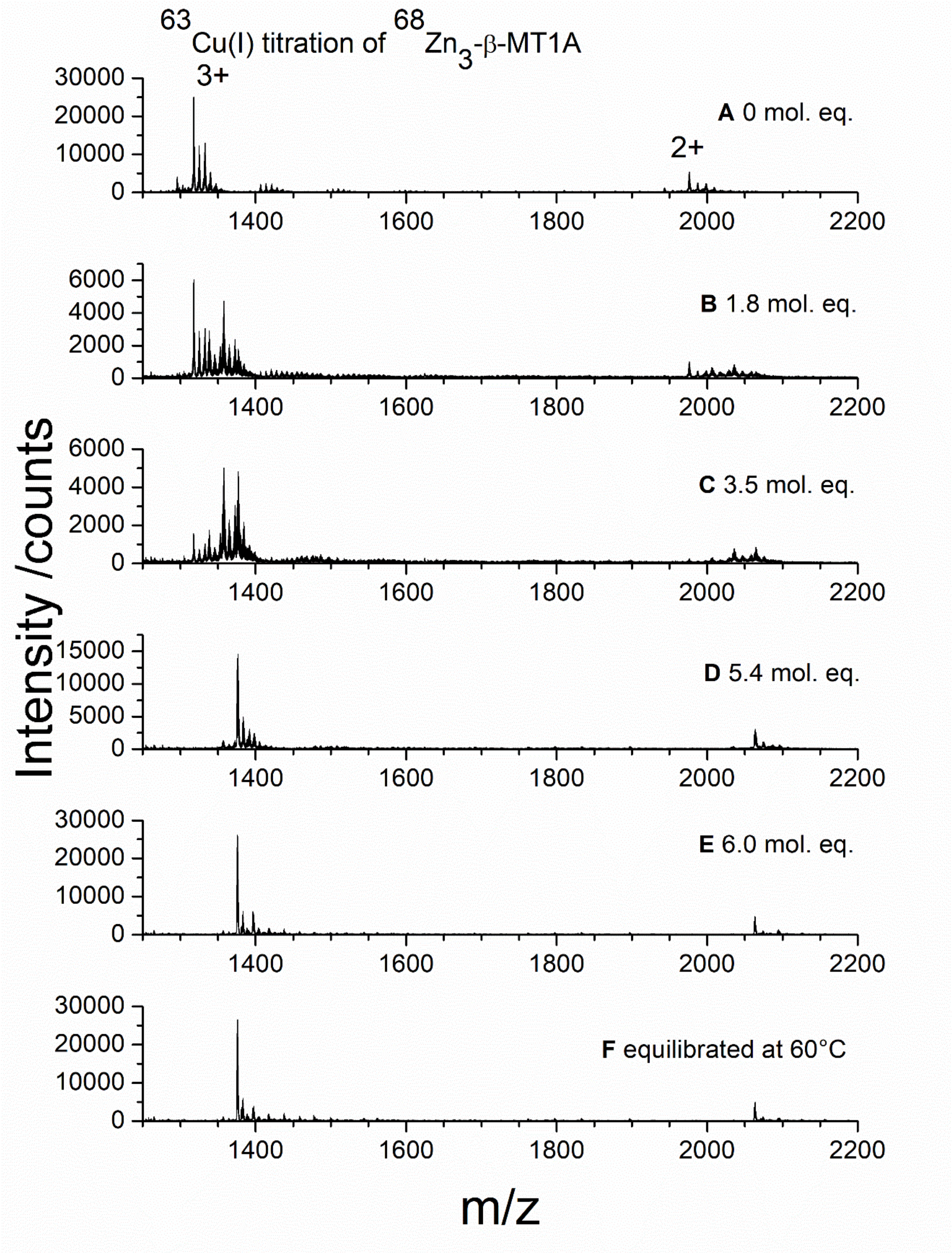

Figure S4 ESI-mass spectral charge state data for the addition of ^{63}Cu(I) ^{68}Zn$_3$ β MT1A at pH 7.4. Typical deconvoluted mass spectral data shown in Figs. 4, S3, and S7.

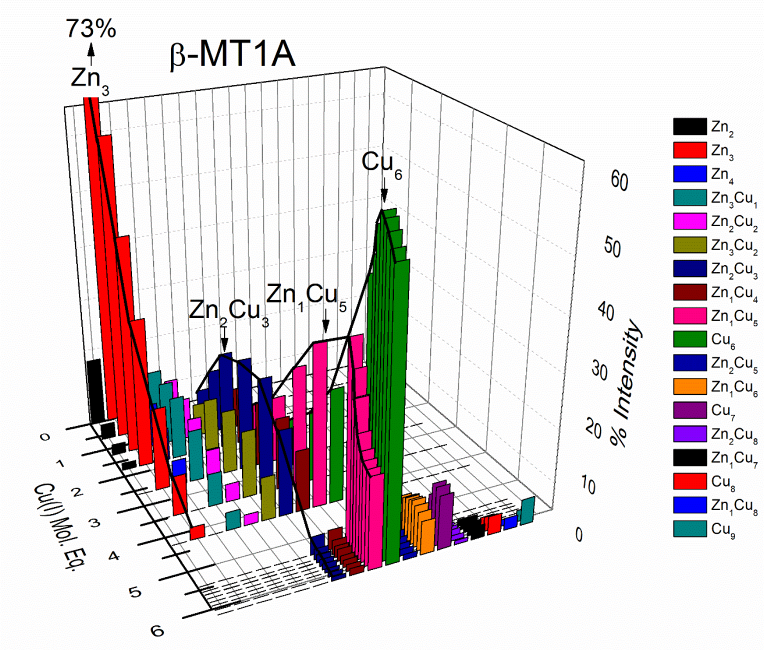

Figure S5 3D speciation diagram showing the species forming after the addition of ^{63}Cu(I) to ^{68}Zn$_3$-β MT1A. Amount of Cu(I) bound to the protein determined from ESI-mass spectra in Fig. 4.

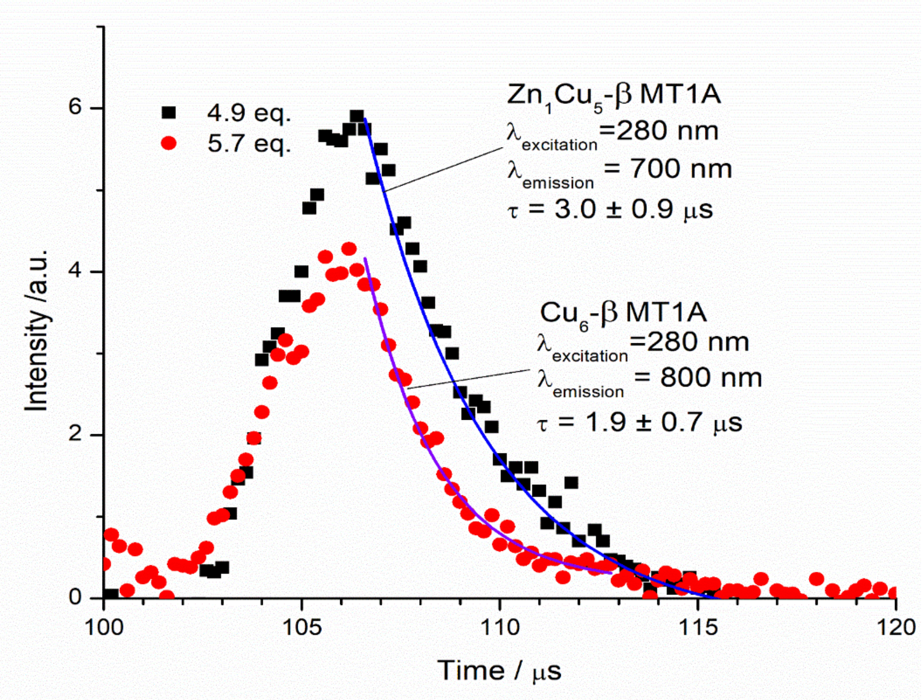

Figure S6 Phosphorescent lifetime data measured after the addition of ^{63}Cu(I) to ^{68}Zn$_3$-β MT1A. λ_{ex} = 280 nm. Phosphorescent emission spectra shown in Fig. 5.

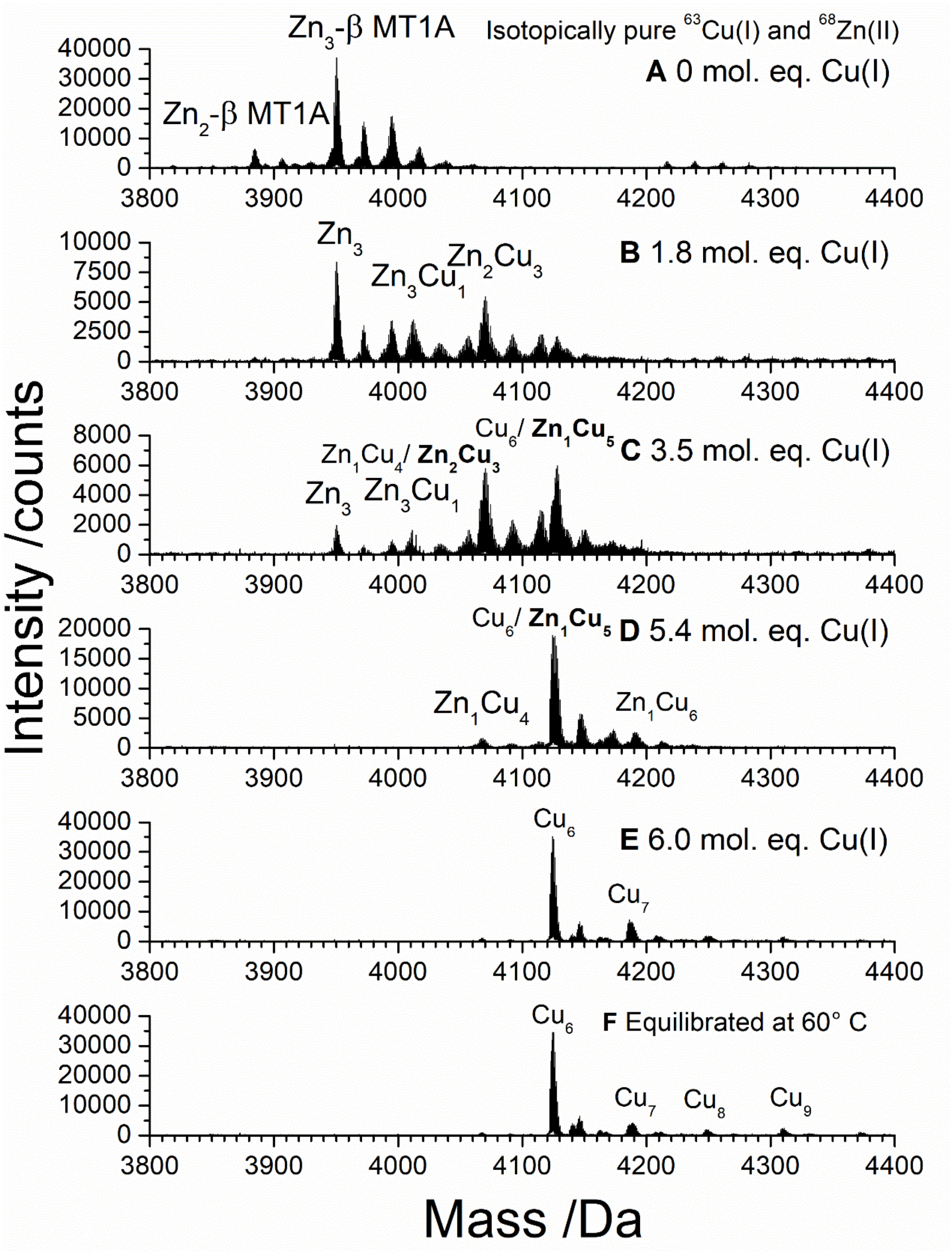

Figure S7 Deconvoluted ESI-mass spectra for Cu(I) addition to Zn_3-β MT1A at pH 7.4 and room temperature (A-E). Despite the addition of excess Cu(I), very little metallation was seen past Cu_6-β MT1A so the sample was heated to 60 °C and equilibrated for two minutes before measuring by ESI-MS again (F).

$^{63}Cu(I)$ Addition to $^{68}Zn_4$-α MT1A at pH 7.4 and room temperature

Table S2: Mass of-species forming upon the addition of $^{63}Cu(I)$ to $^{68}Zn_4$-α MT1A

**Species**	**Mass (Da)**
apo α MT1A	4082
$^{68}Zn_3$-α MT1A	4280
$^{68}Zn_4$-α MT1A	4346
$^{68}Zn_3{}^{63}Cu_1$-α MT1A	4342
$^{68}Zn_2{}^{63}Cu_2$-α MT1A	4338
$^{68}Zn_5$-α MT1A	4412
$^{68}Zn_4{}^{63}Cu_1$-α MT1A	4408
$^{68}Zn_3{}^{63}Cu_2$-α MT1A	4404
$^{68}Zn_2{}^{63}Cu_3$-α MT1A	4400
$^{68}Zn_3{}^{63}Cu_3$-α MT1A	4466
$^{68}Zn_2{}^{63}Cu_4$-α MT1A	4462
$^{68}Zn_1{}^{63}Cu_5$-α MT1A	4458
$^{68}Zn_2{}^{63}Cu_5$-α MT1A	4524
$^{68}Zn_1{}^{63}Cu_6$-α MT1A	4520
$^{68}Zn_3{}^{63}Cu_5$-α MT1A	4590
$^{68}Zn_2{}^{63}Cu_6$-α MT1A	4586
$^{68}Zn_1{}^{63}Cu_7$-α MT1A	4582
$^{68}Zn_3{}^{63}Cu_6$-α MT1A	4652
$^{68}Zn_2{}^{63}Cu_7$-α MT1A	4648
$^{68}Zn_1{}^{63}Cu_8$-α MT1A	4644
$^{68}Zn_2{}^{63}Cu_8$-α MT1A	4710
$^{68}Zn_1{}^{63}Cu_9$-α MT1A	4706
$^{63}Cu_{10}$-α MT1A	4702
$^{68}Zn_1{}^{63}Cu_9$-α MT1A	4768
$^{63}Cu_{11}$-α MT1A	4764

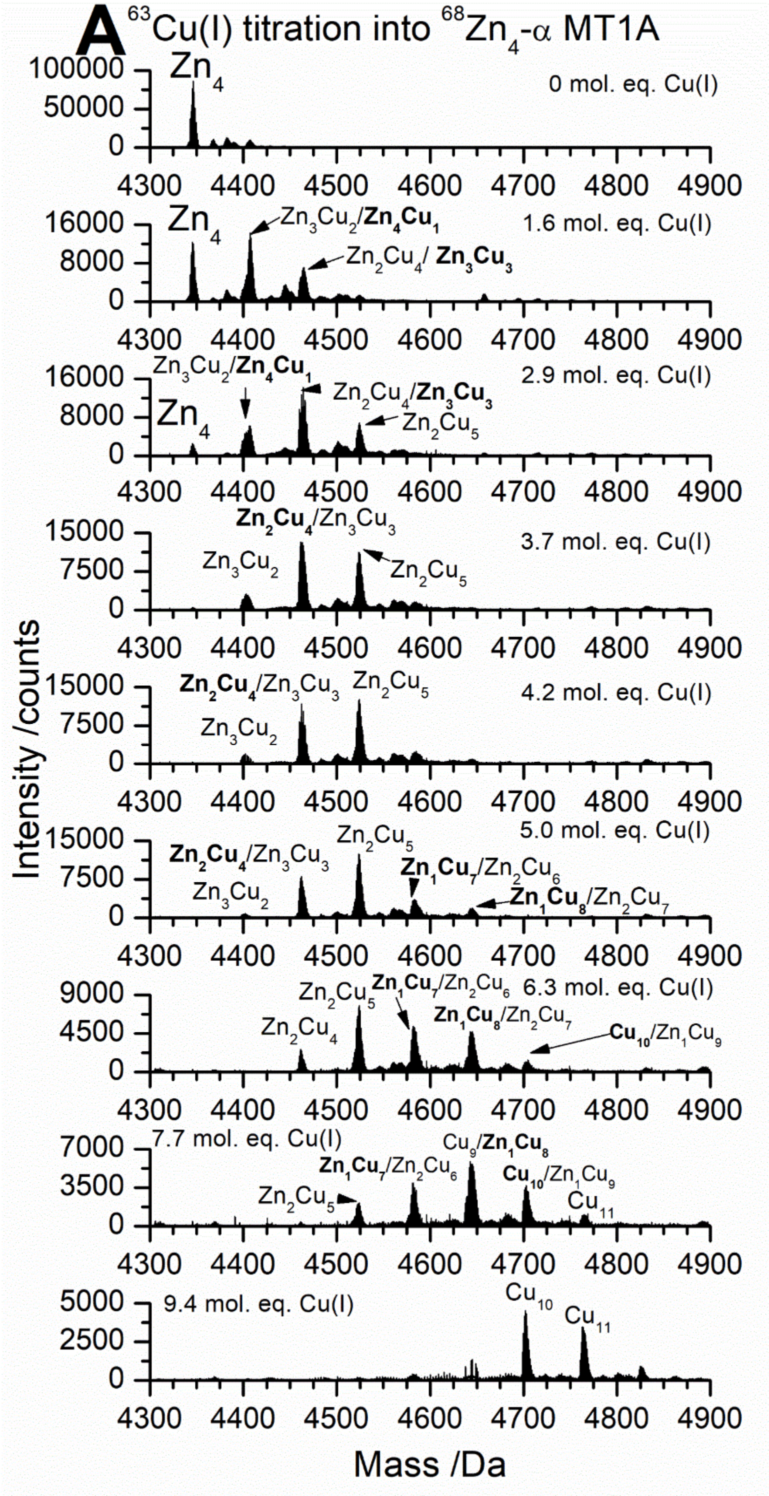

Figure S8 All deconvoluted mass spectra for the ^{63}Cu(I) titration of 35 µM ^{68}Zn$_4$-α MT1A shown in Fig. 7. The titration was carried out at room temperature and pH 7.4.

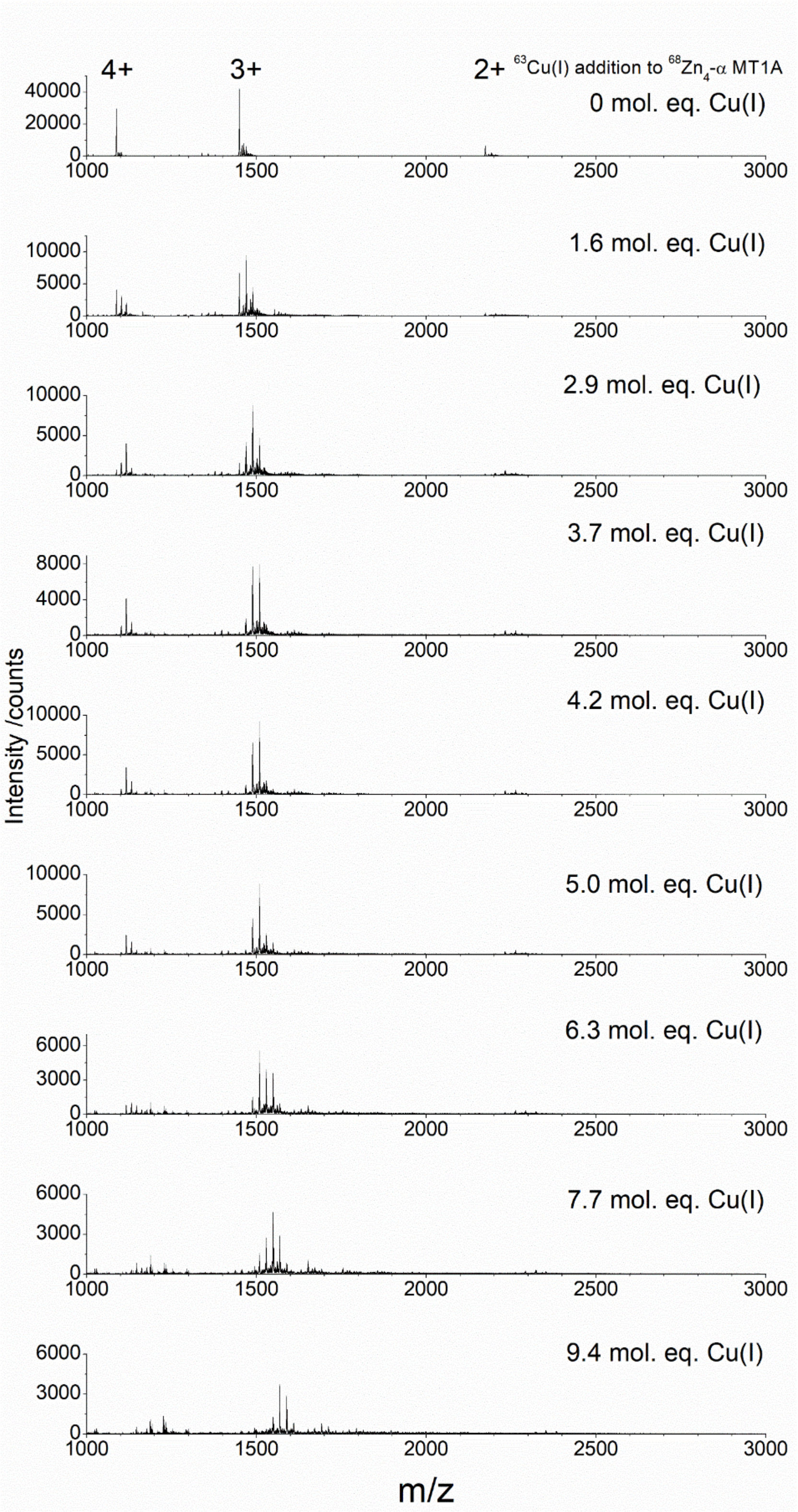

Figure S9 ESI-mass spectral charge state data recorded during the ^{63}Cu(I) titration of 35 µM $^{68}Zn_4$-α MT1A at pH 7.4. Deconvoluted mass spectra shown in Fig. 7 and S8.

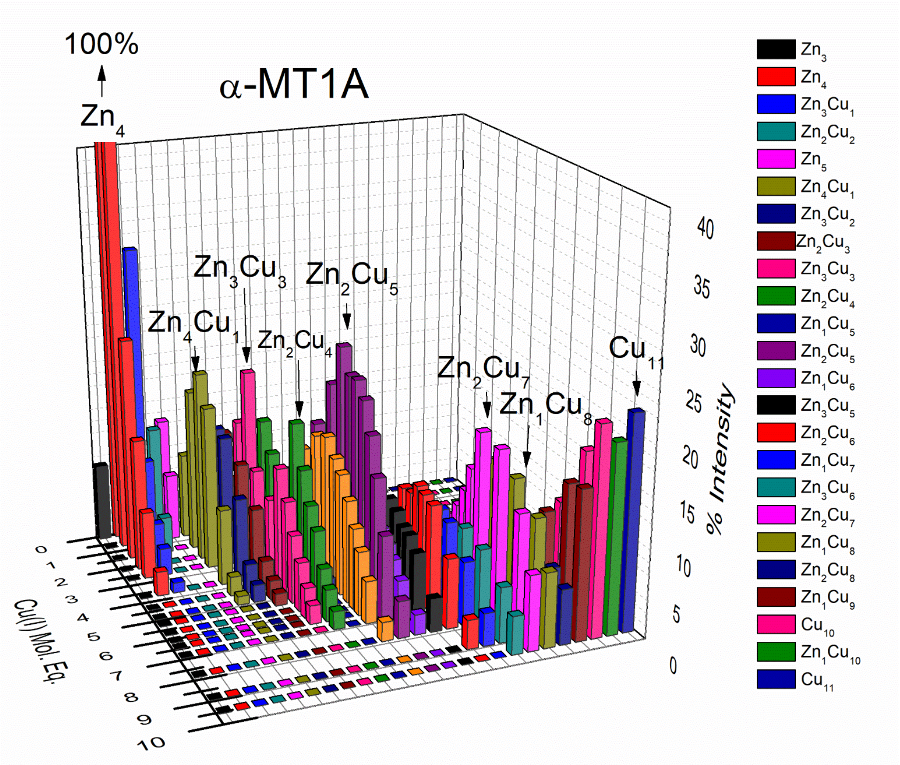

Figure S10 3D speciation diagram showing the species forming after the addition of ^{63}Cu(I) to ^{68}Zn$_4$-α MT1A based on the ESI-mass spectral data shown in Fig. 7.

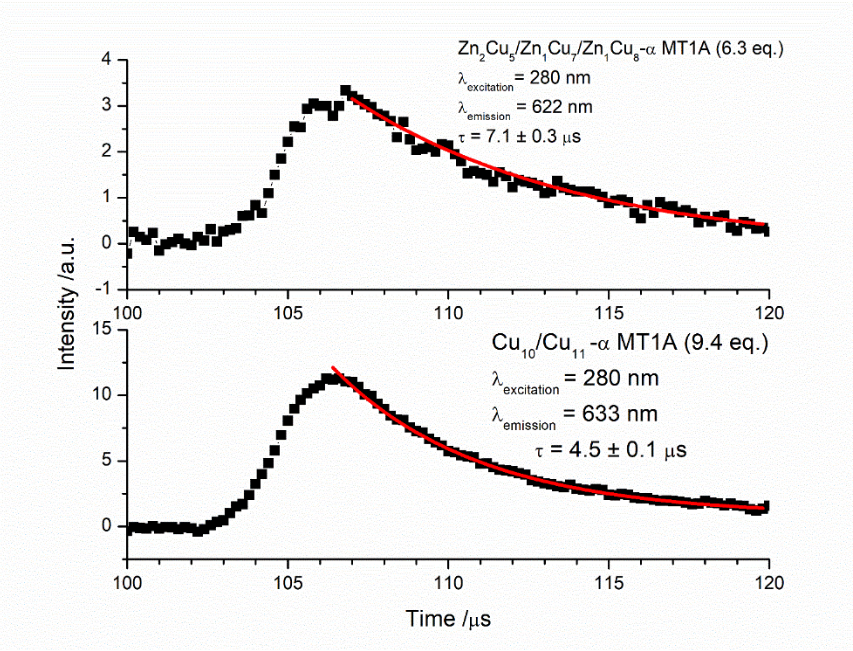

Figure S11 Phosphorescent lifetime data measured after the addition of ^{63}Cu(I) to ^{68}Zn$_4$-α MT1A. λ_{ex} = 280 nm. Phosphorescence spectra shown in Fig. 9.

^{63}Cu(I) Addition to ^{68}Zn$_7$-βα MT1A at pH 7.4 and room temperature

Table S3: Mass of-species forming upon the addition of $^{63}Cu(I)$ to $^{68}Zn_7$-βα MT1A

Species	Mass (Da)
apo βα MT1A	7404
$^{68}Zn_7$-βα MT1A	7866
$^{68}Zn_7{}^{63}Cu_1$-βα MT1A	7928
$^{68}Zn_6{}^{63}Cu_2$-βα MT1A	7924
$^{68}Zn_6{}^{63}Cu_3$-βα MT1A	7986
$^{68}Zn_5{}^{63}Cu_4$-βα MT1A	7982
$^{68}Zn_6{}^{63}Cu_4$-βα MT1A	8048
$^{68}Zn_5{}^{63}Cu_5$-βα MT1A	8044
$^{68}Zn_4{}^{63}Cu_6$-βα MT1A	8040
$^{68}Zn_6{}^{63}Cu_5$-βα MT1A	8110
$^{68}Zn_5{}^{63}Cu_6$-βα MT1A	8106
$^{68}Zn_4{}^{63}Cu_7$-βα MT1A	8102
$^{68}Zn_3{}^{63}Cu_8$-βα MT1A	8098
$^{68}Zn_5{}^{63}Cu_7$-βα MT1A	8168
$^{68}Zn_4{}^{63}Cu_8$-βα MT1A	8164
$^{68}Zn_3{}^{63}Cu_9$-βα MT1A	8160
$^{68}Zn_2{}^{63}Cu_{10}$-βα MT1A	8156
$^{68}Zn_4{}^{63}Cu_9$-βα MT1A	8226
$^{68}Zn_3{}^{63}Cu_{10}$-βα MT1A	8222
$^{68}Zn_2{}^{63}Cu_{11}$-βα MT1A	8218
$^{68}Zn_3{}^{63}Cu_{11}$-βα MT1A	8284
$^{68}Zn_2{}^{63}Cu_{12}$-βα MT1A	8280
$^{68}Zn_3{}^{63}Cu_{12}$-βα MT1A	8346
$^{68}Zn_2{}^{63}Cu_{13}$-βα MT1A	8342
$^{68}Zn_1{}^{63}Cu_{14}$-βα MT1A	8338
$^{68}Zn_2{}^{63}Cu_{14}$-βα MT1A	8404
$^{68}Zn_1{}^{63}Cu_{15}$-βα MT1A	8400
$^{63}Cu_{16}$-βα MT1A	8396
$^{68}Zn_1{}^{63}Cu_{16}$-βα MT1A	8462
$^{63}Cu_{17}$-βα MT1A	8458
$^{68}Zn_1{}^{63}Cu_{17}$-βα MT1A	8524
$^{63}Cu_{18}$-βα MT1A	8520
$^{63}Cu_{19}$-βα MT1A	8582
$^{63}Cu_{20}$-βα MT1A	8644

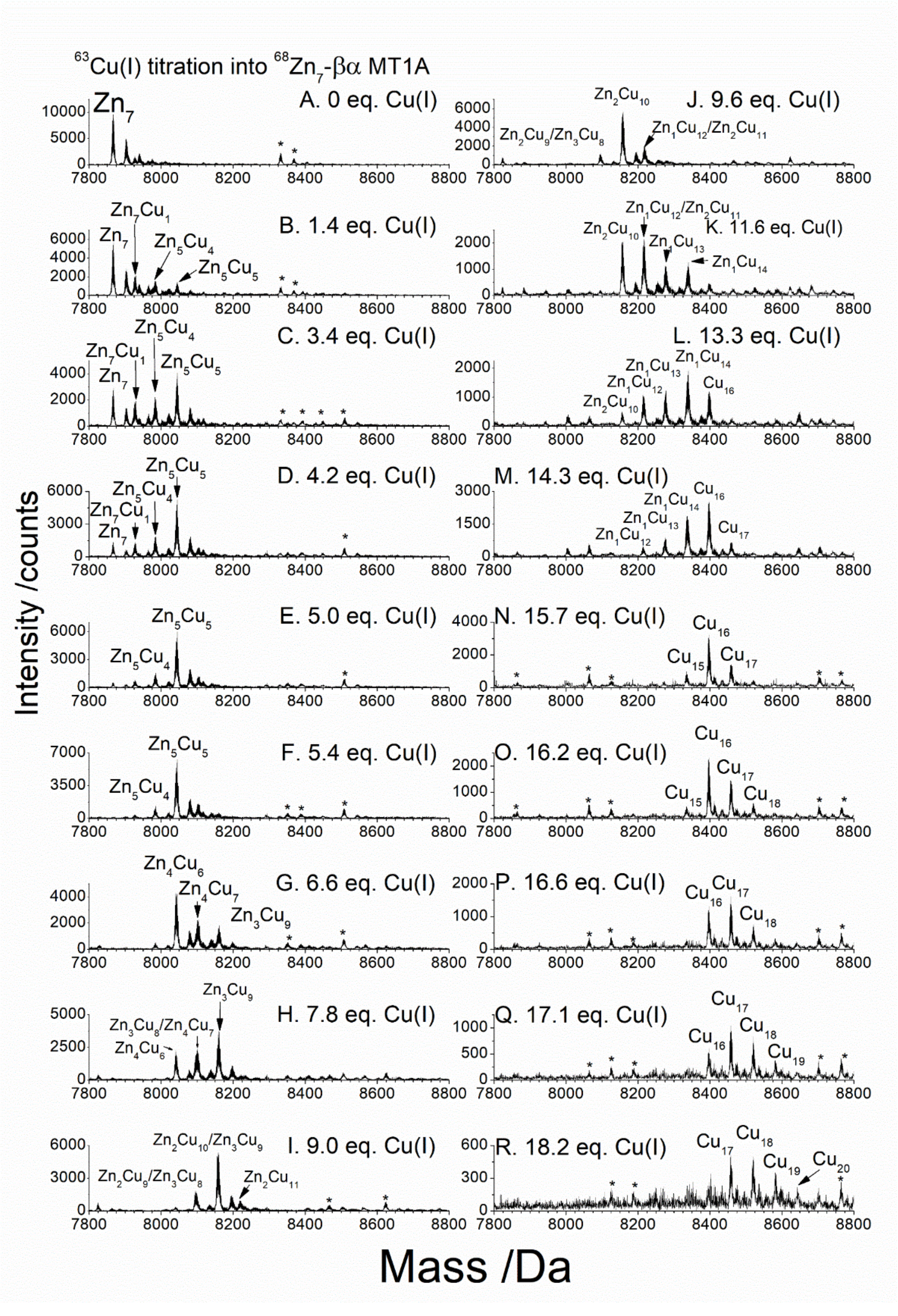

Figure S12 All ESI-mass spectra for the ^{63}Cu(I) titration of 13.6 µM ^{68}Zn$_7$-βα MT1A shown in Fig. 10. The titration was carried out at room temperature and pH 7.4.

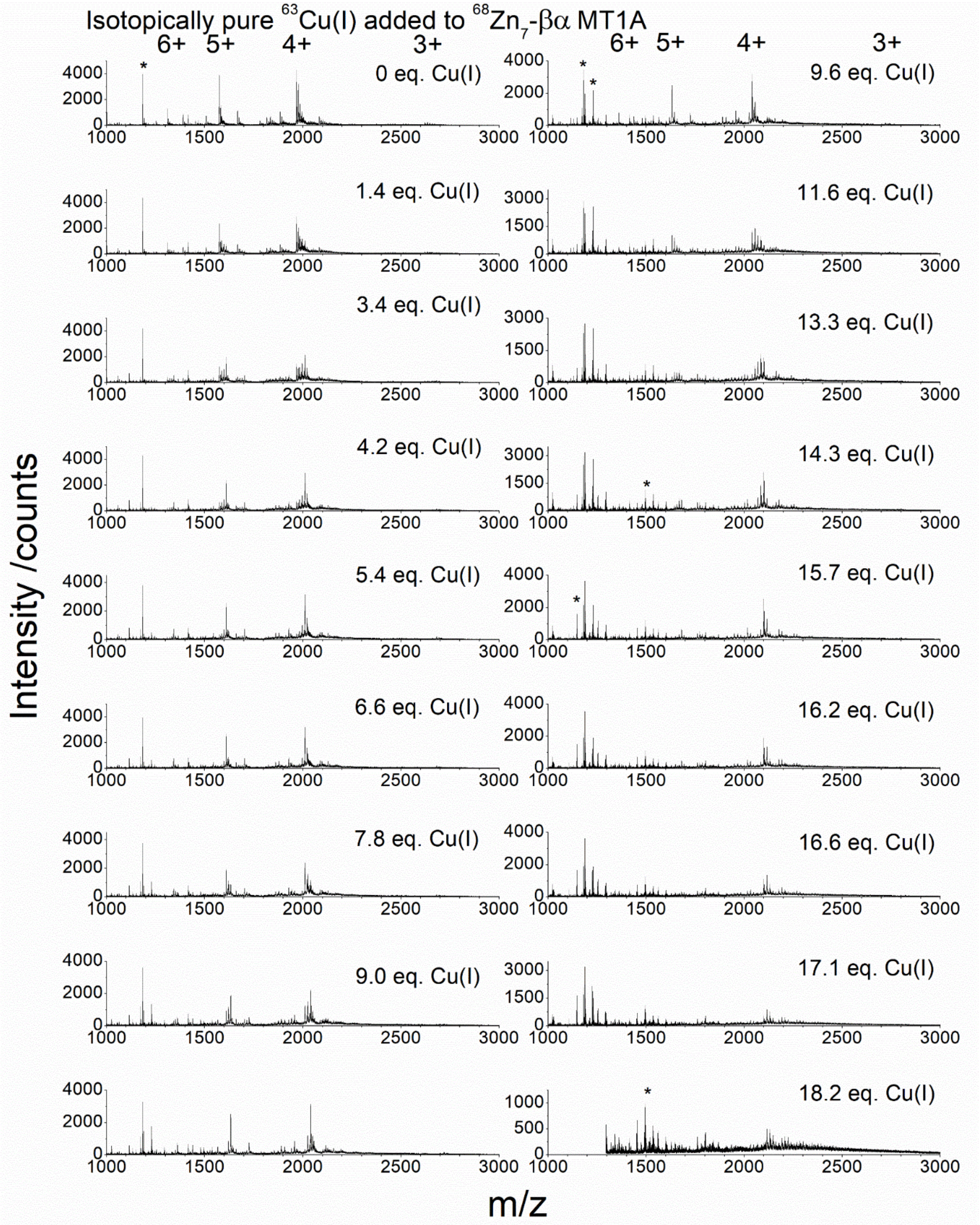

Figure S13 ESI-mass spectral charge state data measured for the stepwise addition of ^{63}Cu(I) into 13.6 µM $^{68}Zn_7$-βα MT1A at pH 7.4. Deconvoluted ESI-mass spectra shown in Fig. S12 and Fig. 10.

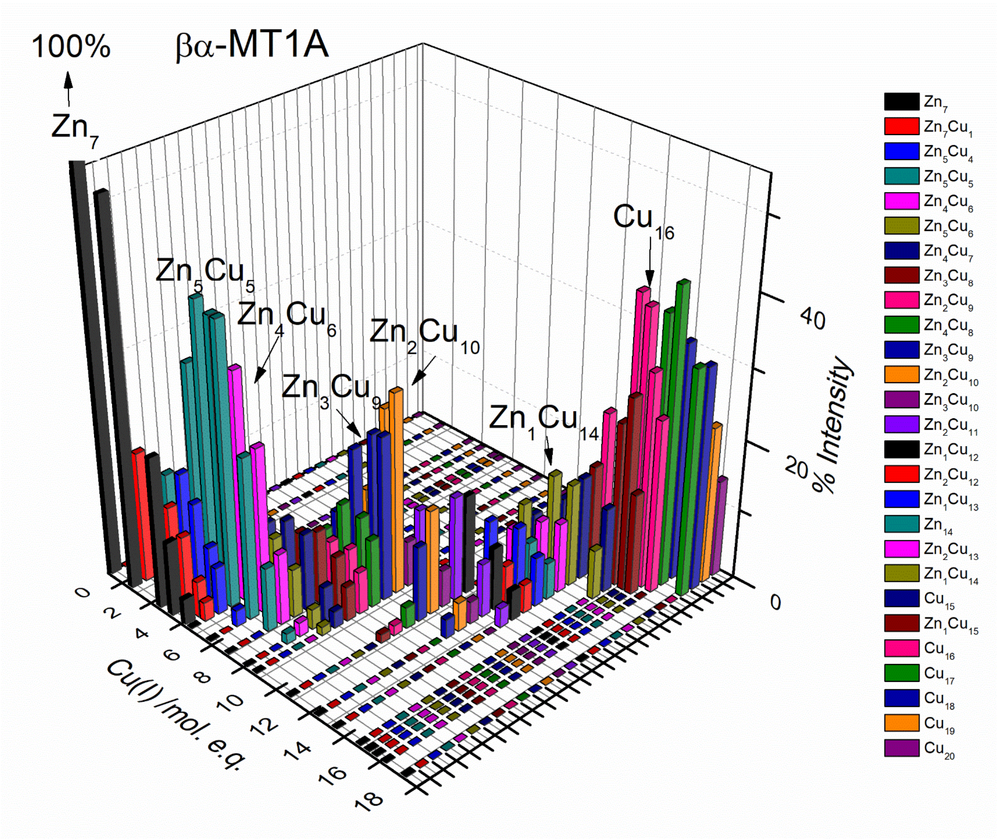

Figure S14 3D speciation diagram showing the species forming after the addition of ^{63}Cu(I) to ^{68}Zn$_7$-βα MT1A based on the deconvoluted ESI-mass spectra shown in Fig. 10.

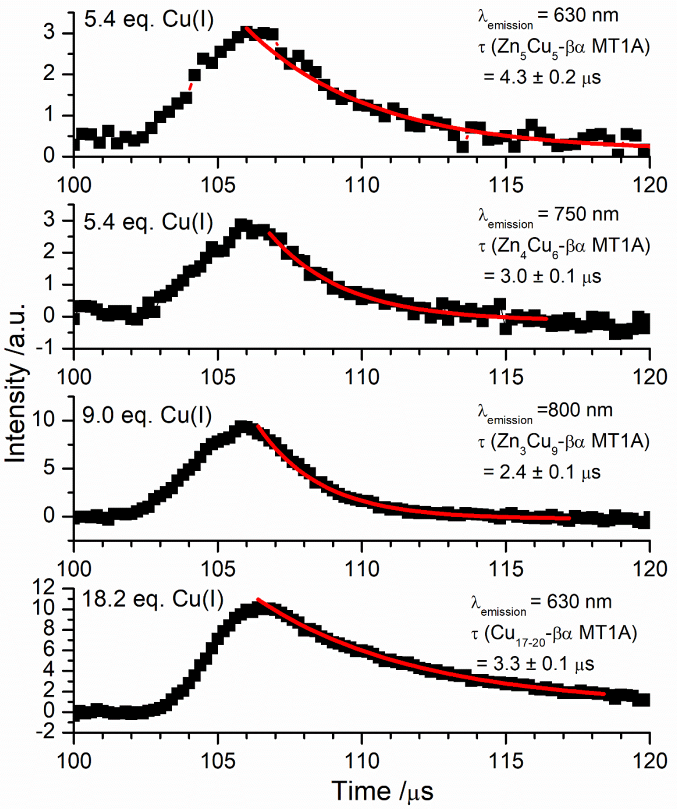

Figure S15 Phosphorescent lifetime data measured after the addition of $^{63}Cu(I)$ to $^{68}Zn_7$-βα MT1A. λ_{ex} = 280 nm. Phosphorescent emission spectra shown in Fig. 11.

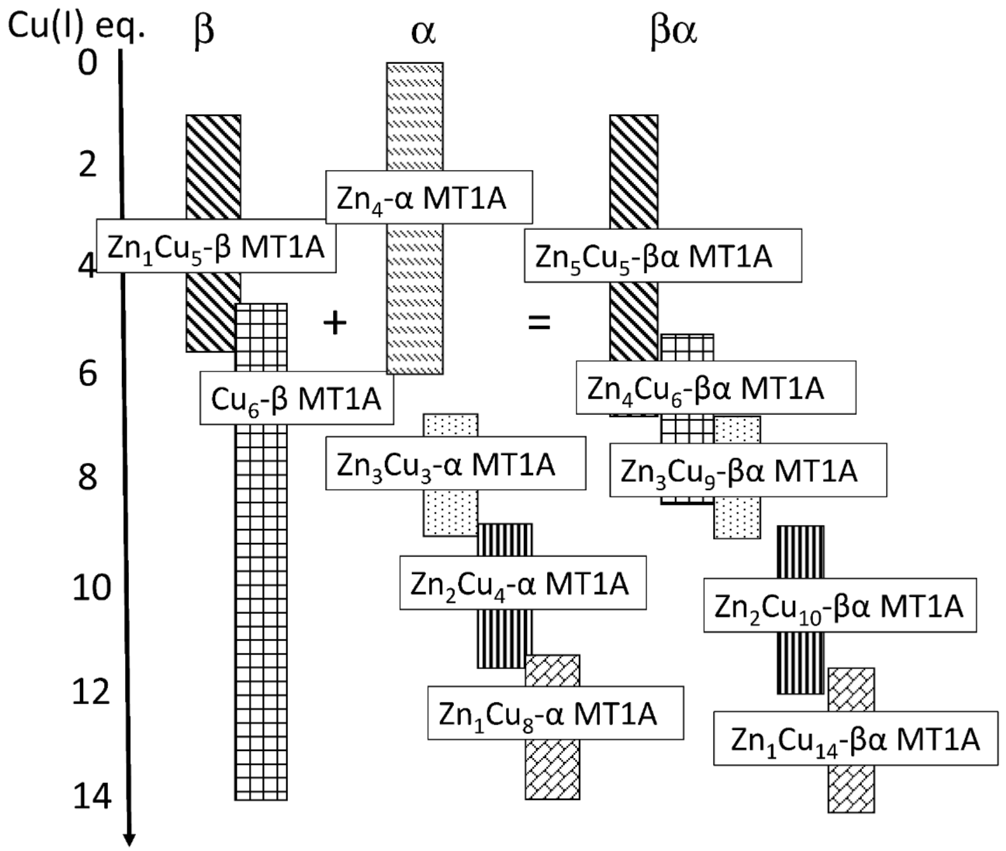

Figure S16 Key species forming from the addition of Cu(I) to Zn_3-β MT1A, Zn_4-α MT1A, and Zn_7-βα MT1A. The species in the β MT1A and α MT1A domain fragments form in the full βα protein to result in the 4 key βα MT1A species.

<u>The total cationic charge is surprisingly constant while Cu(I) is added to Zn(II)-MT</u>

Analysis of the cationic charge of the metals bound to βα-MT1A reveals that most species have a combination of metals that sums to a charge of 14+ to 16+ (Fig. S17) with only one species having a cationic charge of < 14+. Fig. S17 is a chart that shows the cationic charge for Zn_mCu_n-βα MT1A species with different combinations of m and n. The numbers for m and n are shown in black and go across the left side and top of the chart, respectively. Underneath each βα cationic charge are the β and α domain cationic charges for the Zn_aCu_b-β MT1A and Zn_cCu_d-α MT1A species that when combined together, result in the βα MT1A species. The numbers for the β domain a and b values are shown in blue across the left side and top, respectively. The numbers for the α domain c an d values are shown in red across the left side and top, respectively. We assume that the β domain is metallated with Cu(I) up to Cu_6-β before the α domain binds Cu(I). Therefore, the charges of the β domain species have been combined with the charge of Zn_4-α. The charge of the α domain species are combined with the charge of the fully metalled Cu_6-β. The ellipse indicates a region of stability where the species have charges of 14+ to 17+. The box at the bottom of the chart reveals the ranges of cationic charges for the species found in βα MT1A, β MT1A, and α MT1A.

For example, the species, Zn_5Cu_5-βα MT1A, has a cationic charge of +15. It is a combination of Zn_1Cu_5 in the β domain which has a charge of +7, and Zn_4 in the α domain, which has a charge of +8.

Cu (βα)		0	1	2	3	4	5	6	7	8	9	10	11	12	13	14	15	16	17	18	19	20
Cu (β)		0	1	2	3	4	5	6														
Cu(α)								0	1`	2	3	4	5	6	7	8	9	10	11	12		
Zn (βα)	Zn (β) + Zn_4-α																					
7		14	15																			
	3	6+8	7+8																			
6				14	15	16	17															
	2			6+8	7+8																	
5						14	15	16	17													
	1					6+8	7+8															
	Cu_6-β + Zn (α)																					
4								14	15	16	17											
	4							6+8	6+9													
3										14	15	16	17	18								
	3									6+8	6+9	6+10	6+11	6+12								
2											13	14	15	16	17	18	19					
	2										6+7	6+8	6+9	6+10	6+11	6+ 12						
1				Species			Resulting Charge									16	17	18	19			
	1			Zn_7MT + 0-20 Cu^+			14+ -> 20+									6+ 10	6+11	6+12				
0				Zn_3 beta MT + 0-6 Cu^+			6+ -> 7+											16	17	18	19	20
	0			Zn_4 alpha MT + 0-11 Cu^+			8+ -> 12+											6+10	6+11			

Figure S17 Total cationic charge of each Zn,Cu-MT species forming. The total cationic charge from the metals in the β and α domain fragments is added together with the assumption that the β domain fills first forming the species shown in Figs. 4 while the α domain remains as Zn_4-α MT1A. The second half of the charges are calculated based on the β domain fragments remaining as Cu_6 and the α domain forming the species observed in Fig. 7. The sum of the isolated domain charges adds up to the charge seen in βα MT1A at every corresponding species.
